# Association of wearable device-measured vigorous intermittent lifestyle physical activity with mortality

**DOI:** 10.1038/s41591-022-02100-x

**Published:** 2022-12-08

**Authors:** Emmanuel Stamatakis, Matthew N. Ahmadi, Jason M. R. Gill, Cecilie Thøgersen-Ntoumani, Martin J. Gibala, Aiden Doherty, Mark Hamer

**Affiliations:** 1 Charles Perkins Centre, Faculty of Medicine and Health, The University of Sydney, Sydney, New South Wales Australia; 2 School of Cardiovascular and Metabolic Health, University of Glasgow, Glasgow, UK; 3 Danish Centre for Motivation and Behaviour Science, Department of Sports Science and Clinical Biomechanics, University of Southern Denmark, Odense, Denmark; 4 Department of Kinesiology, McMaster University, Hamilton, Ontario Canada; 5 Big Data Institute, Nuffield Department of Population Health, University of Oxford, Oxford, UK; 6 Institute Sport Exercise Health, Division Surgery Interventional Science, University College London, London, UK

**Keywords:** Risk factors, Cardiovascular diseases, Epidemiology

## Abstract

Wearable devices can capture unexplored movement patterns such as brief bursts of vigorous intermittent lifestyle physical activity (VILPA) that is embedded into everyday life, rather than being done as leisure time exercise. Here, we examined the association of VILPA with all-cause, cardiovascular disease (CVD) and cancer mortality in 25,241 nonexercisers (mean age 61.8 years, 14,178 women/11,063 men) in the UK Biobank. Over an average follow-up of 6.9 years, during which 852 deaths occurred, VILPA was inversely associated with all three of these outcomes in a near-linear fashion. Compared with participants who engaged in no VILPA, participants who engaged in VILPA at the sample median VILPA frequency of 3 length-standardized bouts per day (lasting 1 or 2 min each) showed a 38%–40% reduction in all-cause and cancer mortality risk and a 48%–49% reduction in CVD mortality risk. Moreover, the sample median VILPA duration of 4.4 min per day was associated with a 26%–30% reduction in all-cause and cancer mortality risk and a 32%–34% reduction in CVD mortality risk. We obtained similar results when repeating the above analyses for vigorous physical activity (VPA) in 62,344 UK Biobank participants who exercised (1,552 deaths, 35,290 women/27,054 men). These results indicate that small amounts of vigorous nonexercise physical activity are associated with substantially lower mortality. VILPA in nonexercisers appears to elicit similar effects to VPA in exercisers, suggesting that VILPA may be a suitable physical activity target, especially in people not able or willing to exercise.

## Abstract (web summary)

Using data from wearable activity monitors, this study shows that short bursts of physical activity that are done as part of daily life activities and that are only 1 or 2 minutes in length are associated with lower mortality, similar to the effects of regular exercise.

## Main

Physical activity is associated with reduced mortality risk^1^, and reduced risk of CVD^1^ and certain cancers^2–4^. Recently updated guidelines^4,5^, based mostly on questionnaire-derived evidence, recommend 150–300 min of moderate-intensity activity or 75–150 min of vigorous-intensity physical activity (≥6 metabolic equivalents) per week. New emphasis is placed on ‘all activity counts’ occurring across all life domains and regardless of bout duration. This recommendation contrasts with previous guidelines^6,7^ that did not recognize the health value of physical activity bouts lasting <10 min. Besides, little evidence supports the previous guideline because questionnaires can typically capture only longer bouts (for example, ≥10 min) of physical activity and often concentrate on leisure time activities such as gym-based exercise, running and sports^8,9^.

The health effects of each time unit of physical activity are intensity dependent^10–13^. For a given volume of physical activity, higher contributions of VPA are associated with additional mortality risk reduction^10–13^. This is partly due to the enhanced cardiorespiratory adaptations it causes^14^ and the protection it offers against the development of certain cancers^15,16^. Although vigorous-intensity physical activity is time-efficient, vigorous structured exercise-based sessions (for example, gym-based, sports, high-intensity interval training) are not feasible or appealing to the majority of middle-aged adults, as indicated by the very low participation rates^8,9,17^. Over a median follow-up of 3.1 years, a previous UK Biobank accelerometry study^18^ concluded that moderate- to vigorous-intensity activity was associated with greater reductions in all-cause mortality risk than lower-intensity activity. However, VPA was not specifically quantified in this study^18^.

VILPA^19^ refers to brief and sporadic (for example, up to 1 or up to 2 min long) bouts of vigorous-intensity physical activity done as part of daily living, such as bursts of very fast walking while commuting to work or moving from place to place, or stair climbing^20^. No cohort study has examined the associations of VILPA with mortality or other prospective outcomes. For most adults, VILPA may be more feasible than structured exercise because it requires minimal time commitment and involves no specific preparation, equipment or access to facilities. Many common activities of daily living are likely to elicit relative vigorous-intensity effort in physically inactive adults with poor fitness who do not habitually exercise^21^, which is the majority demographic in many countries^8,9,22,23^.

In contrast to questionnaires, wearable devices such as wrist^24,25^ or thigh^26,27^ accelerometers continuously record movement at a high resolution allowing them to capture fine-grain patterns of brief physical activity bouts, such as VILPA^19^. The rapidly growing use of wearable devices in research^25,27–30^ and among consumers^31^ offers opportunities to better understand the health-enhancing potential of VILPA and analogous unexplored movement ‘micro-patterns’. Such potential is greatly enhanced by the recent application of machine learning^32–34^ in studies using wearable devices to understand the health effects of movement.

In a sample of UK Biobank participants with accelerometry data who reported no exercise in their leisure time, we examined the dose–response curves and minimum VILPA dose (daily duration and bout frequency) associated with all-cause, CVD and cancer mortality risk. To provide a population-wide context for our findings, we also examined the dose–response associations of (exercise or nonexercise) VPA with the same mortality outcomes among exercisers in the UK Biobank accelerometry substudy.

## Results

### Description of the study sample

**Fig. 1:**
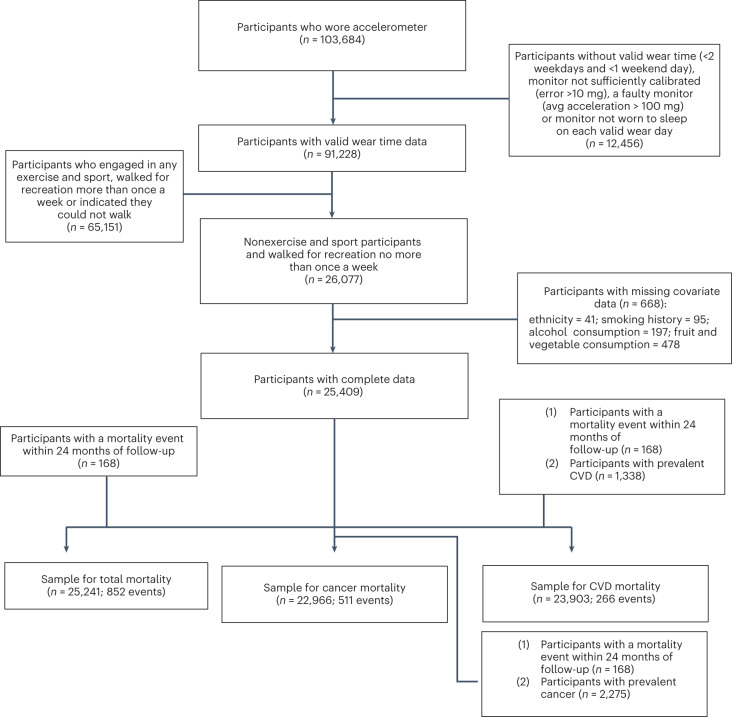
Flow diagram of non-exercisers. Flow diagram of UK Biobank participants for the dose–response analyses of VILPA.

Figure 1 shows the sample derivation process, which resulted in 25,241 (all-cause mortality analyses)/23,903 (CVD mortality analyses)/22,699 (cancer mortality analyses) UK Biobank participants being included in the corresponding analyses. Table 1 presents the characteristics of the sample by daily VILPA frequency. The mean (s.d.) age of participants was 61.8 (7.6) years, and 56.2% were female. Over a mean follow-up of 6.9 (0.8) years (175,528 person-years), 852 deaths were recorded (266 due to CVD and 511 due to cancer).

**Table 1:** Sample characteristics, as stratified by average daily frequency of VILPA bouts (*n* = 25,241)

	Number of daily VILPA bouts lasting up to 2 minutes				
	0	1–2	3–4	≥4	Overall
n	2,816	8,088	8,768	5,569	25,241
Follow-up, years	6.8 (1.0)	6.9 (0.8)	6.9 (0.8)	6.9 (0.7)	6.9 (0.8)
Age, mean (s.d.)	64.7 (6.8)	62.9 (7.4)	61.3 (7.6)	59.7 (7.7)	61.8 (7.6)
Male, *n* (%)	888 (31.5)	2,977 (36.8)	4,079 (46.5)	3,119 (56.0)	11,063 (43.8)
Ethnicity, *n* (%)					
Asian	30 (1.1)	93 (1.1)	119 (1.4)	84 (1.5)	326 (1.3)
Black	18 (0.6)	74 (0.9)	89 (1.0)	80 (1.4)	261 (1.0)
Mixed	16 (0.6)	40 (0.5)	55 (0.6)	46 (0.8)	157 (0.6)
Other	21 (0.7)	76 (0.9)	66 (0.8)	60 (1.1)	223 (0.9)
White	2,731 (97.0)	7,805 (96.5)	8,439 (96.2)	5,299 (95.2)	24,274 (96.2)
Smoking history, *n* (%)					
Current	328 (11.6)	766 (9.5)	775 (8.8)	456 (8.2)	2,325 (9.2)
Previous	1,042 (37.0)	2,898 (35.8)	3,100 (35.4)	1,905 (34.2)	8,945 (35.4)
Never	1,446 (51.3)	4,424 (54.7)	4,893 (55.8)	3,208 (57.6)	13,971 (55.4)
Body mass index	29.6 (5.9)	28.4 (5.3)	27.3 (4.7)	26.3 (4.3)	27.6 (5.1)
Alcohol consumption, *n* (%)^a^					
Never	138 (4.9)	345 (4.3)	300 (3.4)	190 (3.4)	973 (3.9)
Ex-drinker	140 (5.0)	307 (3.8)	266 (3.0)	155 (2.8)	868 (3.4)
Within guidelines	1,760 (62.5)	4,939 (61.1)	5,087 (58.0)	3,203 (57.5)	14,989 (59.4)
Above guidelines	778 (27.6)	2,497 (30.9)	3,115 (35.5)	2,021 (36.3)	8,411 (33.3)
Education, *n* (%)					
College	975 (35.3)	2,941 (37.0)	3,196 (37.2)	2,002 (36.7)	9,114 (36.8)
A/AS level	380 (13.8)	1,010 (12.7)	1,107 (12.9)	702 (12.9)	3,199 (12.9)
O level	621 (22.5)	1,763 (22.2)	1,955 (22.7)	1,209 (22.2)	5,548 (22.4)
CSE	105 (3.8)	376 (4.7)	444 (5.2)	360 (6.6)	1,285 (5.2)
NVQ/HND/HNC	147 (5.3)	461 (5.8)	557 (6.5)	401 (7.4)	1,566 (6.3)
Other	535 (19.4)	1,389 (17.5)	1,337 (15.6)	776 (14.2)	4,037 (16.3)
Fruit and vegetable consumption, *n* (%)^b^					
High	889 (31.6)	2,517 (31.1)	2,693 (30.7)	1,689 (30.3)	7,788 (30.9)
Moderate	1,287 (45.7)	3,766 (46.6)	4,067 (46.4)	2,593 (46.6)	11,713 (46.4)
Low	640 (22.7)	1,805 (22.3)	2,008 (22.9)	1,287 (23.1)	5,740 (22.7)
Family history of CVD, *n* (%)	1,680 (59.7)	4,588 (56.7)	4,752 (54.2)	2,919 (52.4)	13,939 (55.2)
Family history of cancer, n (%)	748 (26.6)	2,109 (26.1)	2,221 (25.3)	1,366 (24.5)	6,444 (25.5)
Medication, *n* (%)					
Cholesterol	720 (25.6)	1,613 (19.9)	1,339 (15.3)	678 (12.2)	4,350 (17.2)
Blood pressure	891 (31.6)	1,859 (23.0)	1,639 (18.7)	740 (13.3)	5,129 (20.3)
Insulin	54 (1.9)	87 (1.1)	65 (0.7)	28 (0.5)	234 (0.9)
Self-rated health, *n* (%)					
Poor	240 (8.5)	491 (6.1)	350 (4.0)	142 (2.5)	1,223 (4.8)
Fair	868 (30.8)	1,969 (24.3)	1,845 (21.0)	1,030 (18.5)	5,712 (22.6)
Good	1,433 (50.9)	4,630 (57.2)	5,290 (60.3)	3,432 (61.6)	14,785 (58.6)
Excellent	259 (9.2)	965 (11.9)	1,270 (14.5)	956 (17.2)	3,450 (13.7)
Sleep (hours per day), median [IQR]	7.3 [6.3, 8.2]	7.3 [6.3, 8.1]	7.4 [6.5, 8.1]	7.4 [6.5, 8.1]	7.4 [6.4, 8.1]
Acceleration magnitude (milli-gravity) [IQR]	20.7 [17.2, 25.3]	24.5 [20.9, 29.3]	28.2 [24.3, 33.3]	33.5 [28.8, 39.6]	27.2 [22.4, 30.6]
Total activity (min per day), median [IQR]^c^	110.2 [70.5, 169.5]	119.1 [82.7, 181.3]	136.3 [99.9, 196.8]	176.8 [133.4, 239.9]	138.3 [96.0, 201.0]
Light activity (min per day), median [IQR]	92.8 [59.0, 145.4]	94.6 [63.8, 146.7]	98.0 [69.0, 146.6]	105.2 [75.4, 149.2]	98.0 [68.0, 147.0]
Moderate activity (min per day), median [IQR]	12.8 [6.4, 24.3]	20.0 [11.4, 33.9]	27.9 [17.3, 44.1]	39.7 [26.0, 60.0]	25.9 [14.6, 43.2]
Vigorous activity (min per day), median [IQR]	–	1.6 [0.9, 2.3]	4.7 [2.6, 5.9]	8.1 [7.3, 9.3]	4.0 [1.3, 9.1]
Percent of total activity in vigorous activity [IQR]	–	0.8 [0.3, 1.7]	3.7 [2.0, 6.1]	7.3 [3.9, 13.8]	3.2 [1.0, 7.9]
VILPA bouts frequency (up to 1 min duration), median [IQR]	–	1 [1, 2]	3 [3, 4]	7 [6, 9]	3 [2, 4]
VILPA bouts frequency (up to 2 min duration), median [IQR]	–	1 [1, 2]	3 [3, 4]	8 [6, 10]	3 [2, 4]
Mortality rate (per 1,000 person-years)					
All-cause mortality	10.4	5.2	4.2	2.6	4.9
CVD mortality^d^	3.1	1.8	1.3	0.5	1.5
Cancer mortality^e^	7.3	3.4	2.8	1.6	3.2

The columns breakdown corresponds to length-standardized VILPA bouts. Values represent mean (s.d.) unless specified otherwise. A/AS level, ; CSE, ; Higher National Certificate, ; Higher National Diploma, ; IQR, interquartile range; National Vocational Qualification, ; O level, .

^1^Alcohol consumption: above guidelines is >14 units per week, where 1 unit = 8 g of ethanol.

^2^Fruits and vegetable consumption: low is <5 servings per day, high is >8 servings per day.

^3^Daily duration of light-, moderate- and vigorous-intensity activity.

^4^Calculated from the CVD mortality sample (*n* = 23,903).

^5^Calculated from the cancer mortality sample (*n* = 22,699).

Supplementary Fig. 1 describes the sample derivation process for the exercisers sample (defined as those who reported any leisure time exercise/sports or more than one recreational walk per week). Over a mean follow-up of 6.9 (0.8) years (432,545 person-years) 62,344 exercisers were included in the all-cause mortality analyses (1,552 events), 56,810 were included in the CVD mortality analyses (303 events) and 56,397 were included in the cancer mortality analyses (736 events). Supplementary Table 1 describes the characteristics of exercisers who, in comparison with the nonexercisers, had higher educational attainment (46.3% versus 36.8% with college/university degree), higher self-rated health (25.2% versus 13.7% with excellent health) and lower medication use (for example, 15.3% versus 20.3% taking blood pressure medication).

### VILPA summary and nonexerciser status

To enable examination of VILPA in our study (brief bouts of nonexercise VPA occurring during daily living), we used information on exercise participation available in the UK Biobank Study (Supplementary Table 2). Our core VILPA analyses only included 25,241 participants who at the UK Biobank baseline (on average 5.5 years before the accelerometry baseline) reported no leisure time exercise participation and no more than one recreational walk per week. For use in sensitivity analyses, we also derived an alternative, more conservative, definition of nonexercisers by excluding participants who reported any recreational walking in addition to any leisure time exercise (*n* = 10,230). A subsample analysis among 2,407 participants of our core sample who had a UK Biobank re-examination an average (s.d.) of 1.5 (1.4) years before the accelerometry measurements showed that the nonexerciser status was stable over time: 82% reported no leisure time physical activity and no more than one recreational walking session per week on both time points. Among the 6,095 entire UK Biobank accelerometry sample participants who reported no exercise at baseline and had a re-examination, 88% maintained their no leisure time physical activity status over time.

In the core VILPA analyses sample of 25,241 participants, almost all VILPA was accrued in bouts lasting up to 1 or up to 2 min: 92.3% of bouts lasted up to 1 min and 97.7% lasted up to 2 min. Excluding VILPA values of zero, the median and maximum VILPA daily duration was 4.0 and 16.0 min per day for both bout lengths; the median and maximum VILPA frequency was 3.0 and 11.0 length-standardized bouts per day. Among the 62,344 exercisers entered in the comparative analyses, the large majority of context-agnostic (exercise or nonexercise) VPA was accrued in bouts lasting up to 2 min (93.1% of all VPA bouts). Median and maximum VPA daily duration was 6.2 and 18.0 min per day; the median and maximum daily frequency was 4.4 and 14.0 length-standardized bouts per day. In the nonexercisers sample, 11.2% of participants recorded no VILPA. In the exercisers sample, 6.9% recorded no VPA.

### Associations of VILPA with all-cause mortality

**Fig. 2:**
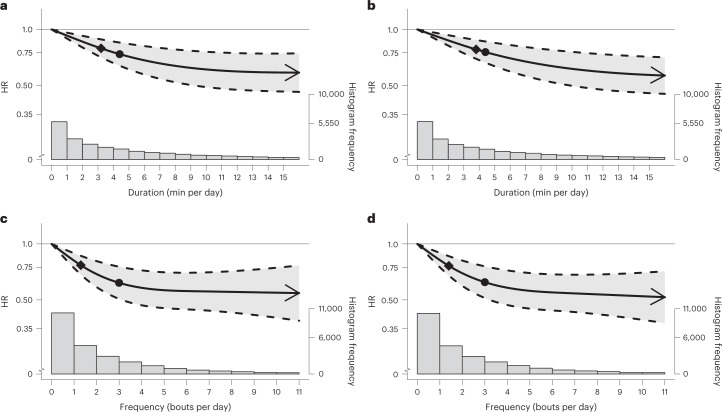
Association of the daily duration and frequency of VILPA with all-cause mortality. **a**,**b**, Dose–response curves showing all-cause mortality HR associated with increasing daily duration of VILPA, for bouts of VILPA up to 1 min (**a**) and 2 min (**b**) in duration. **c**,**d**, Dose–response curves showing all-cause mortality HR associated with increasing daily frequency of VILPA, for length-standardized bouts of VILPA 1 min (**c**) and 2 min (**d**) in duration. Data are shown for *n* = 25,241 participants with 852 events and with a mean follow-up of 6.9 (0.8) years. Diamond, minimal dose, as indicated by the ED_50_ statistic which estimates the daily duration/frequency of VILPA associated with 50% of optimal risk reduction. Circle, HR associated with the median VILPA value (see Supplementary Table 4 for the list of values). Data are adjusted for the covariates listed in the online Methods. The shaded region demarcated by dashed lines represents the 95% CI. The solid line that lies within the shaded region represents the HR. The arrowhead represents the absence of an observed inflection point (for example, larger risk reduction with higher amounts of VILPA). The histogram on the right shows the sample distribution.

In multivariable-adjusted analyses (adjusted for age, sex, light- and moderate-intensity physical activity, longer VPA bouts, smoking, alcohol, sleep duration^35,36^, fruit and vegetable consumption, education, parental history of CVD and cancer, medication use, and prevalent CVD and cancer; Supplementary Table 3), bouts lasting up to 1 min (Fig. 2a,c) and up to 2 min (Fig. 2b,d), exhibited a near-linear dose–response associations of daily VILPA daily duration and frequency with all-cause mortality. Supplementary Table 4A presents the hazard ratio (HR) and 95% confidence intervals (CI) associated with the minimum dose (eliciting 50% of the total effect)^37,38^, and the median and maximum VILPA daily duration and frequency for each bout length. The minimum frequency dose for length-standardized VILPA bouts lasting 1 min was 1.5 bouts per day corresponding to a HR of 0.75 (95% CI 0.66, 0.85). The median and maximum VILPA frequency for length-standardized bouts lasting 1 min were associated with a HR of 0.61 (0.50, 0.74) and 0.52 (0.37, 0.72), respectively. The minimal daily duration dose^37,38^ for VILPA bouts lasting up to 1 min was 3.4 min per day corresponding to a HR of 0.78 (95% CI 0.70, 0.86). The median and maximum VILPA volumes for bouts lasting up to 1 min were associated with a HR of 0.73 (0.63,0.85) and 0.59 (0.46, 0.74). All-cause mortality findings for bouts lasting up to 2 min were similar in terms of the dose–response curves (Fig. 2b,d), the minimal dose values and the magnitude of the associations linked to the median and maximum VILPA daily duration and frequency (Supplementary Table 4A).

### Associations of VILPA with CVD mortality

**Fig. 3:**
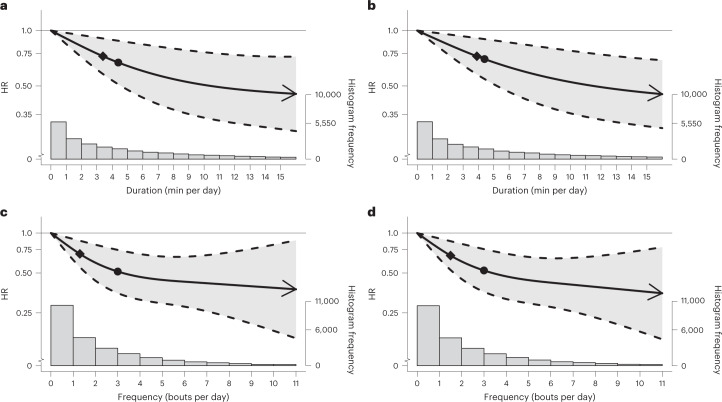
Association of the daily duration and frequency of VILPA with CVD mortality. **a**,**b**, Dose–response curves showing CVD mortality HRs associated with increasing daily duration of VILPA, for bouts of VILPA up to 1 min (**a**) and 2 min (**b**) in duration. **c**,**d**, Dose–response curves showing CVD mortality HRs associated with increasing daily frequency of VILPA, for length-standardized bouts of VILPA1 min (**c**) and 2 min (**d**) in duration. Data are shown for *n* = 23,903 participants with 266 events and with a mean follow-up of 6.9 (0.8) years. Diamond, minimal dose, as indicated by the ED_50_ statistic which estimates the daily duration/frequency of VILPA associated with 50% of optimal risk reduction. Circle, HR associated with the median VILPA value (see Supplementary Table 4 for the list of values). Data are adjusted for the covariates listed in the online Methods. The shaded region demarcated by dashed lines represents the 95% CI. The solid line that lies within the shaded region represents the HR. The arrowhead represents the absence of an observed inflection point (for example, larger risk reduction with higher amounts of VILPA). The histogram on the right shows the sample distribution.

The beneficial associations found in the CVD mortality multivariable-adjusted analyses were more pronounced than the all-cause mortality findings for both bout lengths (Fig. 3a–d and Supplementary Table 4B). For example, the minimum frequency dose for length-standardized VILPA bouts lasting 1 min was 1.4 bouts per day corresponding to a HR of 0.67 (95% CI 0.52, 0.86), and the median and maximum VILPA frequency were associated with a HR of 0.51 (0.35, 0.74) and 0.35 (0.15, 0.81), respectively. The minimal CVD mortality daily duration dose for VILPA bouts lasting up to 1 min was 3.4 min per day corresponding to a HR of 0.73 (95% CI 0.58, 0.91). The median and maximum VILPA daily duration values were associated with a HR of 0.66 (0.50, 0.88) and 0.45 (0.29, 0.72), respectively. CVD mortality findings for bouts lasting up to 2 min were very similar in terms of the dose–response curves (Fig. 3b,d) and all other metrics (Supplementary Table 4B).

### Associations of VILPA with cancer mortality

**Fig. 4:**
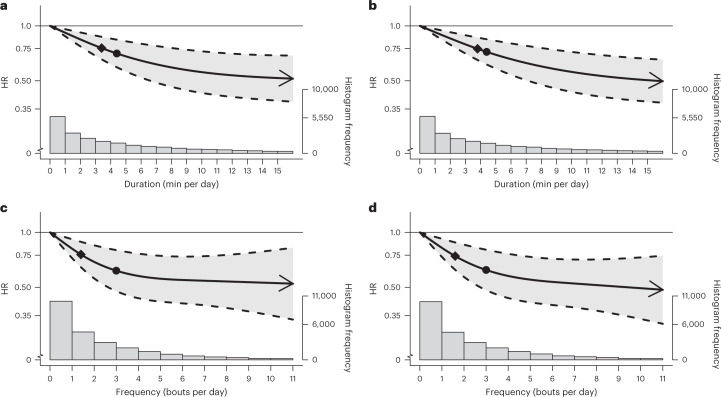
Association of the daily duration and frequency of VILPA with cancer mortality. **a**,**b**, Dose–response curves showing cancer mortality HRs associated with increasing daily duration of VILPA, for bouts of VILPA up to 1 min (**a**) and 2 min (**b**) in duration. **c**,**d**, Dose–response curves showing cancer mortality HRs associated with increasing daily frequency of VILPA, for length-standardized bouts of VILPA 1 min (**c**) and 2 min (**d**) in duration. Data are shown for *n* = 22,966 participants with 511 events and with a mean follow-up of 6.9 (0.8) years. Diamond, minimal dose, as indicated by the ED_50_ statistic which estimates the daily duration/frequency of VILPA associated with 50% of optimal risk reduction. Circle, HR associated with the median VILPA value (see Supplementary Table 4 for the list of values). Data are adjusted for the covariates listed in the online Methods. The shaded region demarcated by dashed lines represents the 95% CI. The solid line that lies within the shaded region represents the HR. The arrowhead represents the absence of an observed inflection point (for example, larger risk reduction with higher amounts of VILPA). The histogram on the right shows the sample distribution.

The findings of the cancer mortality multivariable-adjusted analyses were very consistent with the equivalent all-cause mortality analyses outlined above, in terms of both the dose–response curves (Fig. 4a–d) and the point estimates associated with the minimum dose and the median and maximum VILPA frequency and daily duration values (Supplementary Table 4C). For example, the minimum frequency dose for length-standardized VILPA bouts lasting 1 min was 1.5 bouts per day corresponding to a HR of 0.75 (95% CI 0.63, 0.88). The minimal cancer mortality daily duration dose for VILPA bouts lasting up to 1 min was 3.4 min per day corresponding to a HR of 0.76 (95% CI 0.66, 0.87), whereas the median and maximum VILPA daily duration values were associated with a HR of 0.70 (0.59, 0.84) and 0.51 (0.38, 0.69). Like the other two mortality outcomes, cancer mortality findings for bouts lasting up to 2 min were very similar to bouts lasting up to 1 min across all metrics (Fig. 4a–d and Supplementary Table 4C).

### Sensitivity analyses

**Extended Data Fig. 1:**
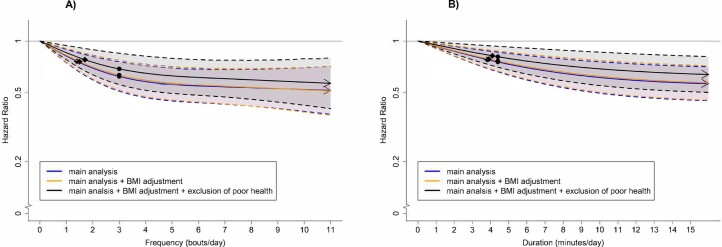
All-cause mortality dose-response of VILPA frequency and duration with adjustment for body mass index and exclusion of poor health. Frequency of length-standardized VILPA bouts up to 2 minutes (**A**) and Duration of VILPA from bouts up to 2 minutes (**B**). Diamond: the minimal dose, as indicated by the ED50 statistic which estimates the daily duration/frequency of VILPA associated with 50% of optimal risk reduction. Circle: the hazard ratio associated with the median VILPA value. Analyses adjusted for age, sex, duration of light intensity physical activity, duration of moderate intensity physical activity, smoking history, alcohol consumption, accelerometer estimated sleep duration, fruit and vegetable consumption, education, self-reported parental history of cardiovascular disease and cancer, and self-reported medication use (cholesterol, blood pressure, and diabetes), and prevalent cancer and CVD. All analyses were additionally adjusted for vigorous physical activity bouts (VILPA frequency analyses) or vigorous physical activity duration (VILPA duration analyses) coming from bouts lasting more than two minutes. Models are sequentially adjusted for: (1) body mass index; (2) exclusion of participants who were underweight (<18.5 kg/m^2^ or self-reported poor health). See Online Methods for further details on the statistical methodology. Shaded region with dashed lines represents 95%CI. Solid line represents point estimate. Arrow represents no inflection point.

**Extended Data Fig. 2:**
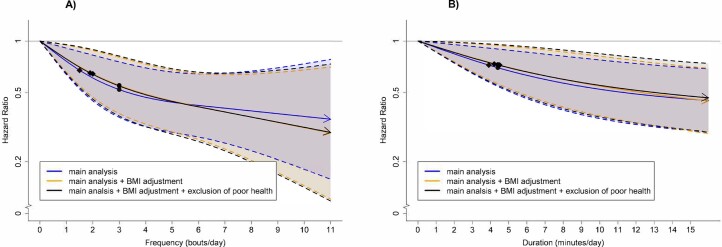
Cardiovascular disease mortality dose-response of VILPA frequency and duration with adjustment for body mass index and exclusion of poor health. Frequency of length-standardized VILPA bouts up to 2 minutes (**A**) and Duration of VILPA from bouts up to 2 minutes (**B**). Diamond: the minimal dose, as indicated by the ED50 statistic which estimates the daily duration/frequency of VILPA associated with 50% of optimal risk reduction. Circle: the hazard ratio associated with the median VILPA value. Analyses adjusted for age, sex, duration of light intensity physical activity, duration of moderate intensity physical activity, smoking history, alcohol consumption, accelerometer estimated sleep duration, fruit and vegetable consumption, education, self-reported parental history of cardiovascular disease and cancer, and self-reported medication use (cholesterol, blood pressure, and diabetes). All analyses were additionally adjusted for vigorous physical activity bouts (VILPA frequency analyses) or vigorous physical activity duration (VILPA duration analyses) coming from bouts lasting more than two minutes. Models are sequentially adjusted for: (1) body mass index; (2) exclusion of participants who were underweight (<18.5 kg/m^2^ or self-reported poor health). See Online Methods for further details on the statistical methodology. Shaded region with dashed lines represents 95%CI. Solid line represents point estimate. Arrow represents no inflection point.

**Extended Data Fig. 3:**
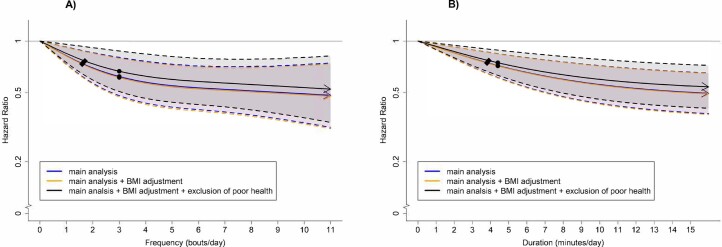
Cancer mortality dose-response of VILPA duration and frequency with adjustment for body mass index and exclusion of poor health. Frequency of length-standardized VILPA bouts up to 2 minutes (**A**) and Duration of VILPA from bouts up to 2 minutes (**B**). Diamond: the minimal dose, as indicated by the ED50 statistic which estimates the daily duration/frequency of VILPA associated with 50% of optimal risk reduction. Circle: the hazard ratio associated with the median VILPA value. Analyses adjusted for age, sex, duration of light intensity physical activity, duration of moderate intensity physical activity, smoking history, alcohol consumption, accelerometer estimated sleep duration, fruit and vegetable consumption, education, self-reported parental history of cardiovascular disease and cancer, and self-reported medication use (cholesterol, blood pressure, and diabetes). All analyses were additionally adjusted for vigorous physical activity bouts (VILPA frequency analyses) or vigorous physical activity duration (VILPA duration analyses) coming from bouts lasting more than two minutes. Models were sequentially adjusted for: (1) body mass index; (2) exclusion of participants who were underweight (<18.5 kg/m^2^ or self-reported poor health). See Online Methods for further details on the statistical methodology. Shaded region with dashed lines represents 95%CI. Solid line represents point estimate. Arrow represents no inflection point.

Excluding participants with poor health (*n* = 1,223) and additionally adjusting for body mass index (Extended Data Figs. 1–3) did not appreciably change the results.

E-values indicated that for our estimates to be null the association of an unmeasured confounder with exposures and mortality should be a HR (lower 95% CI) of 1.87 (1.54) to 3.26 (2.12) for all-cause mortality; 2.10 (1.44) to 5.16 (1.77) for CVD mortality; or 1.97 (1.56) to 3.50 (2.00) for cancer mortality (Supplementary Table 5).

**Extended Data Fig. 4:**
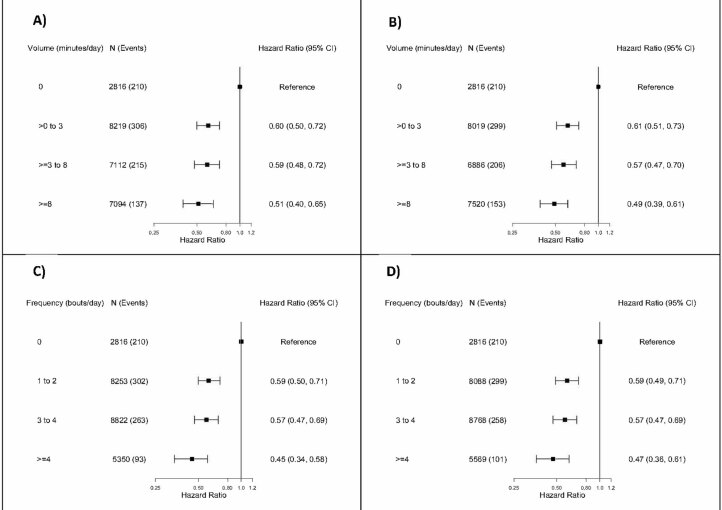
Dose-response association of categorised Vigorous Intermittent Lifestyle Physical Activity (VILPA) duration quarters with all-cause mortality (n = 25,241). Duration of VILPA bout up to 1 minute (**A**); Duration of VILPA bouts up to 2 minutes (**B**); Frequency of length-standardized VILPA bouts up to 1 minute (**C**); and Frequency of length-standardized VILPA bouts up to 2 minutes (**D**). Participants with no VILPA were the reference category. The remaining categories are based on VILPA tertiles. Analyses adjusted for age, sex, duration of light intensity physical activity, duration of moderate intensity physical activity, smoking history, alcohol consumption, accelerometer estimated sleep duration, fruit and vegetable consumption, education, self-reported parental history of cardiovascular disease and cancer, and self-reported medication use (cholesterol, blood pressure, and diabetes), and prevalent cancer and CVD. All analyses were additionally adjusted for vigorous physical activity duration/bouts lasting more than one/two minutes, as appropriate. See Online Methods for further details on the statistical methodology. Sample size = 25,241; events = 868. Error bars represent 95% CI.

Categorical analyses of VILPA daily duration (Extended Data Fig. 4a,b) and frequency (Extended Data Fig. 4c,d) by VILPA tertile-based groups produced results consistent with the main dose–response analyses. Similarly, restricting analyses to those who reported no recreational walking and no leisure time exercise (*n* = 10,230) produced results that were very consistent with the main results in the core (*n* = 25,241) VILPA sample (Supplementary Fig. 2).

### Comparisons between nonexercisers (VILPA) and exercisers (VPA)

**Extended Data Fig. 5:**
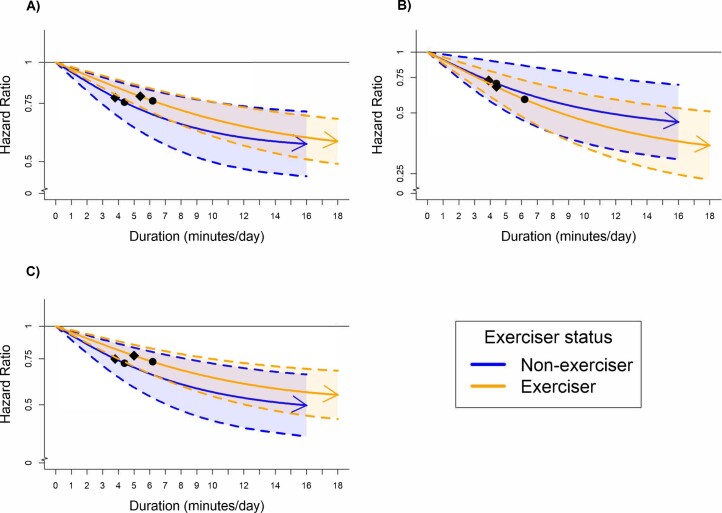
Comparison of the dose-response associations of daily duration of vigorous intermittent lifestyle physical activity (non-exercisers) and context-agnostic (that is exercise or non-exercise) vigorous physical activity (exercisers) with mortality (bouts up to 2 minutes). All-cause mortality (**A**); cardiovascular disease mortality (**B**); and cancer mortality (**C**). Exercisers, n = 62,344 were included in the all-cause mortality (1,552 events), n = 56,810 in the CVD mortality (303 events) and n = 56,397 in the cancer mortality analyses (736 events). Non-exercisers n = 25,241 were included in the all-cause mortality analyses (852 events), were included in the all-cause mortality, n = 23,903 in the CVD mortality analyses (266 events), n = 22,966 in the cancer mortality analyses (511 events). Diamond: the minimal dose, as indicated by the ED50 statistic which estimates the daily duration/frequency of VILPA associated with 50% of optimal risk reduction. Circle: the hazard ratio associated with the median VILPA value. See Supplementary Tables 4 (nonexercisers) and 6 (exercisers) for the list of values. Analyses adjusted for age, sex, duration of light intensity physical activity, duration of moderate intensity physical activity, smoking history, alcohol consumption, accelerometer estimated sleep duration, fruit and vegetable consumption, education, self-reported parental history of cardiovascular disease and cancer, and self-reported medication use (cholesterol, blood pressure, and diabetes), and prevalent cancer and CVD (for all-cause mortality). All analyses were additionally adjusted for vigorous physical activity frequency of bouts lasting more than two minutes. See Online Methods for further details on the statistical methodology. Shaded region with dashed lines represents 95%CI. Solid line represents point estimate. Arrow represents no inflection point.

**Extended Data Fig. 6:**
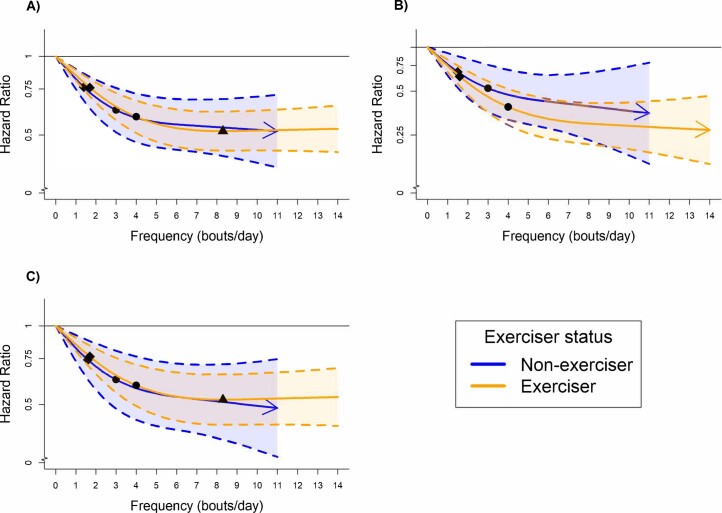
Comparison of the dose-response associations of daily frequency of vigorous intermittent lifestyle physical activity (non-exercisers) and context-agnostic (that is exercise or non-exercise) vigorous physical activity (exercisers) with mortality (length-standardized bouts up to 2 minutes). All-cause mortality (**A**); cardiovascular disease mortality (**B**); and cancer mortality (**C**). Exercisers, n = 62,344 were included in the all-cause mortality (1,552 events), n = 56,810 in the CVD mortality (303 events) and n = 56,397 in the cancer mortality analyses (736 events). Non-exercisers n = 25,241 were included in the all-cause mortality analyses (852 events), were included in the all-cause mortality, n = 23,903 in the CVD mortality analyses (266 events), n = 22,966 in the cancer mortality analyses (511 events). Diamond: the minimal dose, as indicated by the ED50 statistic which estimates the daily duration/frequency of VILPA associated with 50% of optimal risk reduction. Circle: the hazard ratio associated with the median VILPA value. See Supplementary Tables 4 (nonexercisers) and 6 (exercisers) for the list of values. Triangle: optimal dose value defined as the nadir of the curve. Analyses adjusted for age, sex, duration of light intensity physical activity, duration of moderate intensity physical activity, smoking history, alcohol consumption, accelerometer estimated sleep duration, fruit and vegetable consumption, education, self-reported parental history of cardiovascular disease and cancer, and self-reported medication use (cholesterol, blood pressure, and diabetes), and prevalent cancer and CVD (for all-cause mortality). All analyses were additionally adjusted for vigorous physical activity bouts lasting more than two minutes. See Online Methods for further details on the statistical methodology. Shaded region with dashed lines represents 95%CI. Solid line represents point estimate. Arrow represents no inflection point.

Context-agnostic (that is, exercise or nonexercise) VPA in exercisers exhibited an almost identical daily duration and frequency dose–response to VILPA in nonexercisers for all-cause mortality (Extended Data Figs. 5 and 6), with relatively modest differences in minimum dose (4.8 versus 3.4 min per day). No material differences in the CVD and cancer mortality dose–response curves were evident between the two strata and the corresponding minimum doses (Extended Data Figs. 5B,C and 6B,C). Similarly, no appreciable differences existed between exercisers’ and nonexercisers’ VPA/VILPA frequency in terms of their dose–response with the three mortality outcomes (Extended Data Fig. 6). Across all the above analyses there was an almost complete overlap of the 95% CI of the dose–response curves of the two strata/exposures. With very few exceptions, the point estimates associated with the minimum dose and the median VPA frequency and daily duration values (Supplementary Table 6) were also very similar to the equivalent VILPA data (Supplementary Table 4).

Volume analyses based on VILPA (nonexercisers) or VPA (exercisers) energy expenditure (kJ per kg per day) produced evidence of L-shaped associations with all three mortality outcomes, with steeper risk reductions in the lower end of the VILPA/VPA continuum (Supplementary Fig. 3). The only notable exception to this pattern was the VILPA–CVD mortality curve among nonexercisers which indicated a linear association (Supplementary Fig. 3B). Data sparsity and a low number of events at higher levels of the VILPA energy expenditure makes between-strata comparisons and interpretation of these data challenging and less conclusive.

## Discussion

Despite the large health potential of vigorous-intensity physical activity, most adults aged 40 and over do not do vigorous exercise or sports^8,9,17^. Our study is the first investigation, to the best of our knowledge, into the long-term health effects of nonexercise VPA embedded into daily living. We found consistent evidence of beneficial associations of relatively modest VILPA amounts with all-cause, CVD and cancer mortality. VILPA in nonexercisers appeared to elicit beneficial dose–response associations with mortality of similar magnitude to VPA in exercisers, a finding that emphasizes the potential of promoting higher intensity physical activity outside the leisure time exercise domain. Our work has relevance for the development of public health and clinical guidelines because people reporting no structured exercise in leisure time, yet recording VILPA bouts, may be unaware that they are taking short bouts of health-enhancing physical activity of higher intensity. Future guidelines could place emphasis on making people aware that they could potentially experience important benefits from VILPA even though they do not consider themselves to be formal ‘exercisers’.

Although steeper mortality risk reductions occurred at the lower end of the VILPA distribution (up to roughly the median frequency and daily duration values, that is around 3–4 length-standardized bouts per day or 4–5 min per day), there were continuing mortality gains with more VILPA in a near-linear fashion across all three outcomes. With little variation between bouts lasting up to 1 or 2 min and across the three mortality outcomes, a minimum of 3.4–4.1 min of VILPA per day on average was associated with a 22%–28% reduction in mortality risk (compared with not doing VILPA). In terms of minimum daily frequency dose, fewer than two VILPA bouts (lasting 1 or 2 min) were associated with 24%–26% reduction in all-cause and cancer, and a 33% reduction in CVD mortality risk. The median VILPA frequency of 3 length-standardized bouts per day was associated with a 38%–40% reduction in all-cause and cancer mortality and a 48%–49% reduction in CVD mortality risk. The median daily VILPA duration of 4.4 min per day was associated with a 26%–30% reduction in all-cause and cancer mortality and a 32%–34% reduction in CVD mortality risk.

These results are striking but plausible. Proof-of-concept trials^39^ have shown that very small doses of exercise-based intermittent VPA can have rapid and measurable effects on cardiorespiratory fitness, a key causal determinant of CVD^14^. High-intensity interval training^40^ and studies of intermittent stair climbing^39^ have shown that VPA bursts lasting 20 s to a few minutes, performed three to five times a day, can result in substantial improvements in cardiorespiratory fitness in previously inactive adults within a few weeks, providing a plausible physiological basis^14^ for the associations we observed. Our comparisons with exercisers suggest that vigorous exertion is equally important and potentially beneficial for people who choose to be active during leisure time and those less able or willing to do so. Maintained or improved cardiorespiratory fitness owing to vigorous exertion (including VILPA) may partly explain the associations with cancer mortality that we observed: previous observational studies have estimated that a one metabolic equivalent unit higher cardiorespiratory fitness (3.5 ml of oxygen uptake per kg per min) is associated with a 7% reduction in total cancer mortality risk^41^. VPA has also been shown to specifically reduce risk of common cancer sites such as breast^15^, endometrial^16^ and colon^16^.

The 32%–34% lower CVD mortality risk associated with the median VILPA duration of 4.4 min per day (equivalent to just under 31 min of vigorous-intensity physical activity per week) that we observed is comparable with equivalent risk reduction for >75–150 min per week of questionnaire-measured vigorous leisure time physical activity reported in a recent US cohort (36%–45% lower risk compared with no leisure time vigorous activity done in bouts lasting at least 10 min)^11^. This seemingly sizeable difference in VPA amounts associated with a comparable effect size may be explained by the different measurements and domains employed in each study, the select sample of nonexercisers we employed in our study, and the strong possibility that the referent no vigorous leisure time physical activity group in this US study^11^ may do some VILPA. Questionnaires^10–12^ can only capture continuous blocks of time containing a mixture of vigorous activity with interruptions and rest, rather than actual time in vigorous intensity that the wearable devices in our study could quantify. Only one or two in five UK middle-aged adults engage in structured vigorous exercise at least once a month^8,9,17^, suggesting numerous participation barriers. Our findings highlight the potential value of short VPA bursts during daily living to improve overall and cardiovascular health and reduce risk of cancer.

This is the first study of VILPA and prospective health outcomes, using device-based measurement and machine learning-based methods. Although we cannot entirely rule out reverse causation bias, our results were very robust to relevant sensitivity analyses. E-values indicated that unmeasured confounding is unlikely to explain the associations we observed. Although some VILPA activities (for example, carrying heavy shopping bags) may not be perfectly captured by wrist-worn accelerometers, such measurement error is likely random leading to underestimation of the ‘true’ associations with mortality, CVD and cancer. There was a median lag of 5.5 years between the UK Biobank baseline when covariates measurements were taken and the accelerometry study, although covariates were stable over time, with the exception of medication^18^. In addition, adults’ accelerometry-measured physical activity has been shown to be stable over time (for example, >90% of classification accuracy within one quartile over a period of 2–3 years)^42^. The responses to the baseline leisure time physical activity questions (including recreational walking) that formed the basis of our sample selection are subject to measurement error like any other self-reported measure and were also collected 5.5 years before the accelerometry study. However, the nonexerciser status among the UK Biobank accelerometry substudy participants with leisure time physical activity re-examination data was also stable over time (for example, 82%–88% retained the nonexerciser status). The UK Biobank had a very low response rate (5.5%) and it is not representative of the target population^43^. However, recent empirical work has shown that the poor representativeness of the UK Biobank sample does not materially influence the associations between physical activity and mortality outcomes^44^.

In conclusion, we found that as few as two or three short bouts or approximately 3–4 min of VILPA per day were associated with substantially lower all-cause, CVD and cancer mortality risk. Although steeper mortality risk reductions occurred at the lower end of the VILPA distribution, there were continuing gains with larger amounts in a near-linear fashion. Individuals who find structured exercise unappealing or infeasible may consider exploring opportunities to introduce brief but regular bouts of VPA into their daily routines. VILPA in nonexercisers appears to elicit similarly beneficial associations with VPA in exercisers. Future guidelines could emphasize that potentially important health benefits could be accrued through VPA even among people who do not consider themselves to be formal ‘exercisers’. Future trials and device-based cohort studies should further investigate the potential of VILPA (and any-domain VPA in general) as a time-efficient and potentially effective intervention for physically inactive and unfit adults. Our approach shows that wearable devices combined with machine learning-based methods and self-reported information can reveal physical activity “micro-patterns” as targets to prevent premature mortality, CVD and cancer in populations not willing and/or not able to engage in structured exercise during leisure time.

## Methods

### Sample and design

Figure 1 describes the derivation of the analytic sample. The UK Biobank Study is a prospective cohort study of adults aged between 40 and 69 years whose baseline measurements took place between 2006 and 2010. Participants provided informed consent and ethical approval was provided by the UK’s National Health Service, National Research Ethics Service (Ethics Committee reference number: 11/NW/0382).

Between 2013 and 2015 (median 5.5 years after the baseline measurements), 103,684 UK Biobank participants wore a wrist-worn accelerometer for 7 days^24,25^. We excluded participants with missing covariates and insufficient valid wear days. Monitoring days were considered valid if wear time was greater than 16 h. To be included in analysis, participants were required to have at least three valid monitoring days, with at least one of those days being a weekend day^45,46^. We excluded participants who reported that they cannot walk.

To enable examination of VILPA in our study (brief bouts of nonexercise VPA occurring during daily living), we included only participants who reported no leisure time exercise participation and no more than one recreational walk per week. Participation in exercise and recreational walking was measured through a close-ended touch-screen questionnaire that asked participants to report if, how often, and for how long they participate in such activities (Supplementary Table 2). Among the included 14,982 participants who were walking for recreation once a week or less, the average spacing of VILPA bouts was 165.7 (47.0) min within days and 16.7 (5.5) h between days (last session of a day versus first session the day after). The modal median length of the (at most) one and only weekly walking session these participants reported was 30–60 min (32.5% of the 14,982 participants), effectively eliminating the possibility that the device-recorded VILPA bouts occurred during recreational walking.

To provide a comparison between effects of VILPA and (context-agnostic) VPA we repeated the main analyses among ‘exercisers’, defined as those UK Biobank accelerometry substudy participants who did not meet the above criteria to be considered nonexercisers; that is, those who reported any leisure time exercise or more than one recreational walking session per week (Supplementary Table 1).

### Definition of VILPA and choice of bout length

We based the choice of VILPA bout length entered in our analyses on an ongoing study of 58 adults (mean age 55.7 (s.d. 10.1) years) aimed at developing an empirical definition of VILPA (M.N.A., N. Johnson, C.T.-N., M.J.G. and E.S., unpublished data). Participants completed five activities of daily living while wearing an indirect calorimetry unit (Cosmed K5) and Polar heart-rate monitor. The activities included: (1) walking on a flat surface at a self-selected ‘very fast’ pace; (2) walking on a flat surface while carrying shopping-like bags equivalent to 5% of body weight at a self-defined ‘fast’ pace; (3) walking on a flat surface while carrying shopping-like bags equivalent to 10% of body weight at a self-defined ‘fast’ pace; (4) walking at a 2.5% gradient at a self-defined ‘very fast’ pace (treadmill); and (5) walking at a 7.0% gradient at a self-defined ‘very fast’ pace (treadmill). The sequence of activities was randomized for each participant and counterbalanced across participants to prevent biases due to residual fatigue accumulating during the protocol.

Participants performed each activity until vigorous intensity was reached for two of three criteria: (1) %VO_2_max (percentage of maximal oxygen updake) (≥64%); (2) %HRmax (percentage of maximal heart rate) (≥77%); and (3) rating of perceived exertion (Borg scale) ≥15. For %VO_2_max and %HRmax, the threshold had to be met for at least 30 consecutive seconds to minimize the effects of noise. VO_2_max was calculated using the Ebbeling treadmill test and HRmax was calculated using the Tanaka equation^47^. Between activities, participants had 5 min of seated recovery, or until heart rate and breathing returned to resting levels. Resting VO_2_ and heart rate were measured at the beginning of each session with the participant lying supine using 5 min of steady-state (coefficient of variation ≤ 10%). The duration to reach vigorous intensity across all five activities is shown in Supplementary Table 7. As the mean time required to reach vigorous intensity in two of the above three physiological intensity indices was 73.5 s (s.d. 26.2 s) across all activities, we decided to test VILPA bouts lasting up to 1 and up to 2 min in the present analyses. As the length of raw bouts within these two VILPA frequency exposures was highly variable, we length-standardized analytic bouts to one minute (for raw bouts lasting up to 1 minute) or two minutes (for raw bouts lasting up to 2 minutes) using a rolling sum on the time-series data until 1 or 2 minutes, respectively, was reached or exceeded. For example, a participant with five consecutive raw bouts lasting up to 1 minute each (20, 30, 20, 40, and 10 seconds long), would be assigned 1.83 analytic bouts: the first three raw bouts would count as one and the rolling sum would be reset; then the last two raw counts would count as 0.83 length-standardised bouts (50 seconds divided by 60). This bout handling has analytic and interpretational advantages: a) it mitigates against the problem of multicollinearity between raw VILPA frequency and daily VILPA duration, and b) permits a more concrete behavioural interpretation of the VILPA frequency findings than raw bouts, as each length-standardised bout can be specifically interpreted as lasting 1 minute or 2 minutes.

### Wearable device-based physical activity classification

The methods we describe here were used to classify physical activity intensity in both the nonexercisers (main analyses) and exercisers (additional analyses) strata. Supplementary Fig. 4 summarizes how activity intensity was classified using a previously validated random forest (RF) activity classifier^33^. RF is an ensemble of multiple decision trees. Each tree is learned on a bootstrap sample of training data and each node in the tree is split using the best among a randomly selected set of acceleration features. The decisions from each tree are aggregated and a final model prediction is based on majority vote. The RF model requires very little preprocessing of the data because the features do not need to be normalized. In addition, the model is resistant to over-fitting the training data because each tree within the forest is independently grown to maximum depth using a randomly selected subset of features.

This two-stage classifier first categorized physical activity in 10-s windows into one of four activity classes: sedentary, standing utilitarian movements (for example, ironing a shirt, washing dishes), walking activities (for example, gardening, active commuting, mopping floors), running/high energetic activities (for example, active playing with children). These activity classes were then assigned to one of four activity intensities: sedentary, light, moderate and vigorous. Walking activities were classified as light (an acceleration value of <100 mg), moderate (≥100 mg) and vigorous (≥400 mg) intensity^48^. For example, for a VILPA bout lasting up to 2 min, 12 consecutive 10-s windows needed to be classified as vigorous. When there were more than 12 consecutive vigorous activity windows, these bouts counted as long VPA sessions in the corresponding analyses (2.3% of all VPA bouts). Differentiation between sleep^36^ and nonwear^35^ was identified using the change in tilt angle and acceleration standard deviation. Monitors were calibrated^49^ and corrected for orientation^50^ using previously published methods, although residual signal and alignment uncertainties may persist.

Activities in an independent sample of 98 participants (age 56.4 ± 15.7 years ; 53.1% female) from the US^51^ (University of California Irvine Center for Machine Learning and Intelligent Systems Physical Activity Monitoring for Aging People study (published data), accessible at https://archive.ics.uci.edu/ml/datasets) and Australia^52^ (University of Queensland Where and When at Work study (published data) and University of Sydney Intermittent Lifestyle Physical Activity Study (unpublished data)) providing 103,607 activity samples from structured and free-living activities (17,267 min) were used to assess robustness and generalizability of the classifier (Supplementary Tables 8 and 9). For free-living activities participant-worn or researcher-held Go-Pro video-recordings were used to attain ground-truth physical activity. Video files were imported into the Noldus Observer XT software v16.0 for continuous direct observation coding. A two-stage direct observation scheme was implemented in which the participant’s movement behavior was coded for activity type and then activity intensity based on the Compendium of Physical Activities^53^. The direct observation system generated a vector of date–time stamps corresponding to the start and finish of each movement event, which were used to assign the activity codes to the corresponding time segments of the accelerometer data. Interobserver reliability was assessed by dual coding. The intraclass correlation coefficient for coding activities was 0.91 (0.87–0.94).

Performance was further evaluated in a separate sample of 151 adults (age range 18–91 years, 65.6% female; Supplementary Fig. 5) recruited from the UK^34^ (University of Oxford Capture 24 study (published data), accessible at https://ora.ox.ac.uk/objects/uuid:99d7c092-d865-4a19-b096-cc16440cd001). Participants in this data set wore body cameras that provided pictures every 20 s to annotate ground-truth free-living activity labels. The picture-based activity coding scheme has been previously described^34^. A total of 172,360 activity samples (28,727 min) were provided by participants.

### Outcome ascertainment

Because of the nature of rolling updates for the data linkage, participants were followed up to 31 October 2021, with deaths obtained through linkage with the National Health Service (NHS) Digital of England and Wales or the NHS Central Register and National Records of Scotland. CVD mortality was defined as death attributed to diseases of the circulatory system, excluding hypertension, diseases of arteries and lymph (ICD-10 codes: I0, I11, I13, I20–I51, I60–I69). Cancer mortality was defined as death attributed to any cancer excluded in situ, benign, uncertain, nonmelanoma skin cancer or non-well-defined cancers (ICD-10 codes beginning ‘C0’, ‘C1’, ‘C2’, ‘C3’, ‘C4’ (excluding C49.9), ‘C5’, ‘C6’, ‘C70’, ‘C71’, ‘C72’, ‘C73’, ‘C74’, ‘C75’, ‘C7A’, ‘C8’ or ‘C9’).

### Statistical analyses

In our study, the range of VILPA values (and context-agnostic VPA values in exercisers) was capped at the 97.5 percentile to minimize the influence of sparse data. To reduce the possibility of reverse causation through prodromal/undiagnosed disease, all analyses excluded those with an event within the first 2 years of follow-up. We also excluded those with prevalent CVD and prevalent cancer at baseline (CVD and cancer mortality analyses, respectively).

We examined the dose–response of average daily duration and frequency of VILPA bouts lasting up to 1 min and up to 2 min using Cox proportional hazards (all-cause mortality) and Fine–Gray subdistribution hazards to account for competing mortality risks (CVD and cancer mortality)^54^. In all analyses, we set knots at the 10th, 50th and 90th percentiles. Departure from linearity was assessed by a Wald test. Proportional hazards assumptions were tested using Schoenfeld residuals in the models with all three outcomes and no violations were observed (all *P* > 0.05). Analyses were adjusted for age, sex, daily duration of light- and of moderate-intensity physical activity, mutual adjustment for daily duration and frequency of vigorous-intensity physical activity bouts lasting more than 1 to 2 min as appropriate, smoking, alcohol, accelerometry-estimated sleep duration^35,36^, fruit and vegetable consumption, education, parental history of CVD and cancer, medication use (insulin, blood pressure, cholesterol). All-cause mortality analyses were also adjusted for prevalent CVD and cancer, CVD analyses were adjusted for prevalent cancer, and cancer analyses were adjusted for prevalent CVD (Supplementary Table 3 provides full covariate definitions**)**.

In the exercisers stratum of the UK Biobank accelerometry sample, we repeated the above multivariable-adjusted analyses for daily duration and frequency of (context-agnostic) VPA for bouts lasting up to 2 min, and we compared findings with the equivalent VILPA findings using overlay dose–response plots.

To assert the degree to which VILPA and VPA may contribute to mortality beyond the associations of overall movement volume, we also carried out a volume analysis based on energy expenditure using methods analogous to the study by Strain et al.^18^ We calculated physical activity energy expenditure for all VILPA and VPA bouts lasting up to 2 min.

To provide conservative point estimates we calculated the ‘minimal dose’, defined as VILPA volume/frequency associated with 50% of the optimal risk reduction^37,38^. We also present point estimates (HRs and 95% CI) associated with the median and maximum volume/frequency VILPA values. We calculated E-values to estimate the plausibility of bias from unmeasured confounding^30,55^.

We conducted sensitivity analyses of VILPA with additional adjustment for body mass index. To investigate potential reverse causation bias we also excluded participants who had poor self-rated health. In another sensitivity analysis, we tested the influence of applying a conservative definition of ‘nonexercisers’ by restricting analyses to the 10,230 participants who reported no recreational walking and no leisure time exercise.

We performed all analysis using R statistical software v.4.2.1 with RMS v.6.3.0 and survival package v.3.3.1.

We reported this study as per the Strengthening the Reporting of Observational Studies in Epidemiology guidelines (Supplementary Table 10).

### Reporting summary

Further information on research design is available in the Nature Portfolio Reporting Summary linked to this article.

## Online content

Any methods, additional references, Nature Portfolio reporting summaries, source data, extended data, supplementary information, acknowledgements, peer review information; details of author contributions and competing interests; and statements of data and code availability are available at 10.1038/s41591-022-02100-x.

## Supplementary information

Supplementary Information Supplementary Tables 1–10 and Figs. 1–5.

Reporting Summary

## Data Availability

The UK Biobank data that support the findings of this study can be accessed by researchers on application (https://www.ukbiobank.ac.uk/register-apply/). Variables derived specifically for this study will be returned along with the code to the UK Biobank for future applicants to request. Availability of other datasets related to the study: University of California Irvine Center for Machine Learning and Intelligent Systems Physical Activity Monitoring for Aging People: https://archive.ics.uci.edu/ml/datasets; University of Queensland Where and When at Work study: available upon reasonable request to the study’s PI^11^; University of Sydney Intermittent Lifestyle Physical Activity Study: available upon reasonable request to the authors; University of Oxford Capture 24 study: https://ora.ox.ac.uk/objects/uuid:99d7c092-d865-4a19-b096-cc16440cd001

## Code availability

The statistical code used in the analyses of this manuscript can be downloaded from https://zenodo.org/record/7187927#.Y0ZfoHZBy3A
