# Supplementary material for: Association of wearable device-measured vigorous intermittent lifestyle physical activity with mortality
Source: Nat Med. 2022 Dec 8;28(12):2521–9. doi: 10.1038/s41591-022-02100-x (PMC9800274; doi:10.1038/s41591-022-02100-x)

nature medicine

Article

https://doi.org/10.1038/s41591-022-02100-x

Association of wearable device-measured vigorous intermittent lifestyle physical activity with mortality

In the format provided by the authors and unedited

Device-measured vigorous intermittent lifestyle physical activity and mortality: the UK Biobank

Supplementary Material

Page	Item
3	**Supplementary Table 1:** Exercisers' characteristics by average daily frequency of (exercise or non-exercise) vigorous intensity physical activity (VPA) bouts (n=62,344)
6	**Supplementary Table 2:** Questions to assess participation in leisure time physical activity
7	**Supplementary Table 3:** Covariate definitions
8	**Supplementary Table 4:** Hazard ratios associated with the minimum dose and median and maximum VILPA values among non-exercisers for up to 1 and up to 2 minute bouts
11	**Supplementary Table 5:** E-values for minimum dose, and median/maximum VILPA values for all-cause, cardiovascular disease mortality, and cancer mortality
13	**Supplementary Table 6:** Hazard ratios associated with the minimum dose and median and maximum VPA values among exercisers for up to 2 minute bouts
14	**Supplementary Table 7:** Mean durations to reach vigorous intensity across five activities (unpublished data)
15	**Supplementary Table 8:** Intensity classification performance in 98 US and Australian adults
15	**Supplementary Table 9:** Confusion matrix of activity classification in 98 US and Australian adults
16	**Supplementary Table 10:** STROBE Statement
19	**Supplementary Figure 1:** Flow diagram of exercisers
20	**Supplementary Figure 2:** Dose-response association of daily duration of vigorous intermittent lifestyle physical activity (VILPA) duration of bouts up to 2 minutes among those who reported no leisure time exercise and *no* recreational walking
21	**Supplementary Figure 3:** Dose-response associations of energy expenditure-based volume of vigorous intermittent lifestyle physical activity (non-exercisers) and vigorous intensity physical activity (exercisers) with mortality (bouts lasting up to 2 minutes).

22	**Supplementary Figure 4:** Physical activity type and intensity diagram
23	**Supplementary Figure 5:** Participant-level specific recall and precision of activity classification in 151 UK adults

**Supplementary Table1:** Exercisers’ characteristics by average daily frequency of (exercise or non-exercise) vigorous intensity physical activity (VPA) bouts (n=62,344)					
	**Number of VPA bouts lasting up to two minutes per day**				
	0	1 to 2	3 to 4	>=4	Overall
n	4,281	15,380	21,908	20,775	62,344
Follow up, yrs	6.9 (0.9)	6.9 (0.8)	6.9 (0.7)	7.0 (0.7)	6.9 (0.8)
Age, mean (SD)	65.2 (7.0)	63.1 (7.6)	61.1 (7.8)	58.9 (7.8)	61.1 (7.9)
Male, n (%)	1,249 (29.2)	5,242 (34.1)	9,551 (43.6)	11,012 (53.0)	27,054 (43.4)
Ethnicity, n (%)					
Asian	37 (0.9)	137 (0.9)	222 (1.0)	247 (1.2)	643 (1.0)
Black	21 (0.5)	92 (0.6)	141 (0.6)	187 (0.9)	441 (0.7)
Mixed	14 (0.3)	69 (0.4)	106 (0.5)	129 (0.6)	318 (0.5)
Other	28 (0.7)	111 (0.7)	155 (0.7)	184 (0.9)	478 (0.8)
White	4,181 (97.7)	14,971 (97.3)	21,284 (97.2)	20,028 (96.4)	60,464 (97.0)
Smoking history, n (%)					
Current	299 (7.0)	980 (6.4)	1,264 (5.8)	1,066 (5.1)	3,609 (5.8)
Previous	1,598 (37.3)	5,755 (37.4)	8,033 (36.7)	7,104 (34.2)	22,490 (36.1)
Never	2,384 (55.7)	8,645 (56.2)	12,611 (57.6)	12,605 (60.7)	36,245 (58.1)
Body Mass Index	28.3 (5.2)	27.2 (4.6)	26.2 (4.0)	25.3 (3.6)	26.3 (4.2)
Alcohol consumption, n (%)[1]					
Never	151 (3.5)	460 (3.0)	485 (2.2)	470 (2.3)	1,566 (2.5)
Ex-drinker	157 (3.7)	406 (2.6)	515 (2.4)	443 (2.1)	1,521 (2.4)
Within guidelines	2,680 (62.6)	8,987 (58.4)	12,151 (55.5)	11,213 (54.0)	35,031 (56.2)
Above guidelines	1,293 (30.2)	5,527 (35.9)	8,757 (40.0)	8,649 (41.6)	24,226 (38.9)
Education, n (%)					
College	1,773 (41.4)	6,861 (44.6)	10,117 (46.2)	10,103 (48.6)	28,854 (46.3)
A/AS level	538 (12.6)	2,002 (13.0)	3,020 (13.8)	2,748 (13.2)	8,308 (13.3)

O level	883 (20.6)	3,148 (20.5)	4,285 (19.6)	3,963 (19.1)	12,279 (19.7)
CSE	118 (2.8)	442 (2.9)	753 (3.4)	814 (3.9)	2,127 (3.4)
NVQ/HND/HNC	220 (5.1)	729 (4.7)	1,092 (5.0)	1,036 (5.0)	3,077 (4.9)
Other	749 (17.5)	2,198 (14.3)	2,641 (12.1)	2,111 (10.2)	7,699 (12.3)
Fruit and vegetable consumption, n (%)[2]					
High	1,346 (31.4)	4,784 (31.1)	6,665 (30.4)	6,436 (31.0)	19,231 (30.8)
Moderate	1,920 (44.8)	6,988 (45.4)	9,804 (44.8)	9,147 (44.0)	27,859 (44.7)
Low	1,015 (23.7)	3,608 (23.5)	5,439 (24.8)	5,192 (25.0)	15,254 (24.5)
Family history of CVD, n (%)	2,575 (60.1)	9,050 (58.8)	11,934 (54.5)	10,693 (51.5)	34,252 (54.9)
Family history of cancer, n (%)	1,130 (26.4)	4,023 (26.2)	5,567 (25.4)	4,938 (23.8)	15,658 (25.1)
Medication, n (%)					
Cholesterol	925 (21.6)	2,593 (16.9)	2,778 (12.7)	1,870 (9.0)	8,166 (13.1)
Blood pressure	1,167 (27.3)	3,147 (20.5)	3,177 (14.5)	2,064 (9.9)	9,555 (15.3)
Insulin	51 (1.2)	106 (0.7)	104 (0.5)	84 (0.4)	345 (0.6)
Self-rated health, n (%)					
Poor	149 (3.5)	350 (1.2)	268 (1.2)	146 (0.7)	913 (1.5)
Fair	851 (19.9)	2,459 (16.0)	2,675 (12.2)	1,851 (8.9)	7,836 (12.6)
Good	2,646 (61.8)	9,495 (61.7)	13,427 (61.3)	12,207 (58.8)	37,775 (60.6)
Excellent	625 (14.6)	3,049 (19.8)	5,523 (25.2)	6,558 (31.6)	15,755 (25.2)
Sleep (hrs/day), median [IQR]	7.4 [6.4, 8.2]	7.3 [6.4, 8.1]	7.4 [6.6, 8.1]	7.4 [6.6, 8.1]	7.4 [6.5, 8.1]
Acceleration magnitude [IQR][4]	22.3 [18.6, 26.4]	25.8 [22.0, 30.4]	29.7 [25.5, 35.1]	36.6 [31.2, 44.0]	30.3 [25.0, 37.7]
Total activity (min/day), median [IQR][3]	113.5 [76.3, 170.9]	125.6 [90.0, 188.3]	139.7 [106.9, 185.8]	165.9 [147.4, 232.2]	145.4 [99.7, 211.4]
Light activity (min/day), median [IQR]	94.1 [62.9, 145.3]	98.3 [67.6, 151.5]	99.4 [71.3, 149.9]	109.0 [79.0, 154.3]	102.2 [72.2, 151.5]
Moderate activity (min/day), median [IQR]	14.7 [7.9, 27.3]	22.2 [12.8, 36.3]	30.9 [19.3, 47.7]	42.1 [27.4, 62.4]	30.9 [18.0, 49.7]

Vigorous activity (min/day), median [IQR]	-	1.7 [1.0, 2.4]	5.9 [4.9, 7.7]	9.6 [9.1, 14.0]	6.2 [2.4, 14.3]
Percent of total activity in vigorous activity [IQR]	-	0.8 [0.3, 1.7]	4.8 [3.1, 6.5]	8.2 [6.9, 9.5]	4.9 [1.7, 9.4]
VILPA bouts frequency (up to 1 minute duration), median [IQR]	-	2.[1, 2]	4 [3, 4]	9 [8, 12]	3 [2, 10]
VILPA bouts frequency (up to 2 minutes duration), median [IQR]	-	2 [1, 2]	4 [3, 4]	10 [8, 13]	4 [2, 11]

Values represent mean (SD) unless specified otherwise.
[1]1 unit = 8g, above guidelines > 14 units/week
[2]Fruits and vegetables servings/day, low<5 servings/day, high>8 servings/day
[3] Duration of light, moderate, and vigorous intensity activity
[4]milligravity

Supplementary Table 2: Questions to assess participation in leisure time physical activity

Types of physical activity in last 4 weeks UK Biobank Field ID: 6164:	• Walking for pleasure (not as a means of transport)<br>• Other exercises (eg: swimming, cycling, keep fit, bowling)<br>• Strenuous sports<br>• Light DIY (eg: pruning, watering the lawn)<br>• Heavy DIY (eg: weeding, lawn mowing, carpentry, digging)<br>• None of the above<br>• Prefer not to answer
Each time you did strenuous sports, about how long did you spend doing it? (UK Biobank field ID: 1001)	• Less than 15 minutes<br>• between 15 and 30 minutes<br>• between 30 minutes and 1 hour<br>• between 1 hour and 1.5 hours<br>• between 1.5 hours and 2 hours between 2 and 3 hours<br>• over 3 hours, do not know<br>• prefer not to answer
How many times in the last 4 weeks did you do strenuous sports? (UK Biobank field ID: 991)	• Once in the last 4 weeks<br>• 2-3 times in the last 4 weeks<br>• Once a week<br>• 2-3 times a week<br>• 4-5 times a week<br>• Every day<br>• Do not know<br>• Prefer not to answer
Each time you did other exercises such as swimming, cycling, keep fit, about how long did you spend doing it? (UK Biobank field ID: 3647)	• Less than 15 minutes<br>• between 15 and 30 minutes<br>• between 30 minutes and 1 hour<br>• between 1 hour and 1.5 hours<br>• between 1.5 hours and 2 hours between 2 and 3 hours<br>• over 3 hours, do not know<br>• prefer not to answer
How many times in the last 4 weeks did you do other exercises such as swimming, cycling, keep fit? (UK Biobank field ID: 3637)	• Once in the last 4 weeks<br>• 2-3 times in the last 4 weeks<br>• Once a week<br>• 2-3 times a week<br>• 4-5 times a week<br>• Every day<br>• Do not know<br>• Prefer not to answer

How many times in the last 4 weeks did you go walking for pleasure? (UK Biobank field ID: 971)	• Once in the last 4 weeks<br>• 2-3 times in the last 4 weeks<br>• Once a week<br>• 2-3 times a week<br>• 4-5 times a week<br>• Every day<br>• Do not know<br>• Prefer not to answer

Supplementary Table 3: Covariate definitions

**Variable**	**Definition**	**UK Biobank field ID (if applicable)**
Age	Continuous	34, 52, accelerometer date-timestamp
Sex	Female/Male	31
Light intensity	Standing utilitarian movements, slow walking (<3 METs)	Derived from accelerometer data (see Online Methods)
Moderate intensity	Brisk walking, energetic activities (≥3 to <6 METs)	Derived from accelerometer data (see see Online Methods)
Frequency/duration of bouts above the specified bout in each frequency/duration analysis	Number of bouts above the specified bout in the analysis. Eg, for the analysis of bouts lasting up to 2 minutes in duration, this variable contained bouts/duration that were more than 2 minutes in duration	Derived from accelerometer data (see see Online Methods)
Smoking status	Never, past, current	20116
Alcohol consumption	Never, ex-drinker, within guidelines, above guidelines	20117, 1558
Sleep duration	Hours spent sleeping	Derived from accelerometer data (see Online Methods)
Diet	Fruits and vegetables servings/day, categorised as low (<5 servings/day), moderate (5 to 8 servings/day) and high (>8 servings/day)	1309, 1319, 1289, 1299
Prevalent cancer	Identified by self-report and cancer registry	20001, 100092
Prevalent CVD	Identified by self-report and hospitalisation	20002, 2000
Education	College/University; A/AS level; O levels; CSE; NVQ/HND/HNC; other	6138

Parental history of CVD	Self-reported mother or father diagnosed with heart disease or stroke	20107, 20110
Use of cholesterol medication	Yes/No	6177, 6153
Use of blood pressure medication	Yes/No	6177, 6153
Use of diabetes medication	Yes/No	6177, 6153

Supplementary Table 4: Hazard ratios associated with the minimum dose and median and maximum VILPA values among non-exercisers for up to 1 and up to 2 minute bouts

A. All-cause mortality

Frequency	Dose	HR	Lower 95 CI	Upper 95 CI
up to 1 min bout minimum dose	1.5	0.75	0.66	0.85
up to 1 min bout VILPA median dose	3.0	0.61	0.50	0.74
up to 1 min bout VILPA maximum dose	11.0	0.52	0.37	0.72
up to 2 min bout minimum dose	1.4	0.76	0.68	0.86
up to 2 min bout VILPA median dose	3.0	0.62	0.51	0.76
up to 2 min bout VILPA maximum dose	11.0	0.52	0.38	0.71
Duration	Dose	HR	Lower 95 CI	Upper 95 CI
up to 1 min bout minimum dose	3.4	0.78	0.70	0.88
up to 1 min bout median VILPA dose	4.4	0.73	0.63	0.85
up to 1 min bout VILPA maximum dose	16.0	0.59	0.46	0.74
up to 2 min bout minimum dose	4.1	0.77	0.68	0.87
up to 2 min bout median VILPA dose	4.4	0.74	0.65	0.85
up to 2 min bout VILPA maximum dose	16.0	0.57	0.45	0.71

Minimal dose (ED50 value): defined as the duration/frequency of VILPA associated with 50% of the optimal risk reduction. The VILPA duration and frequency median values were calculated in the sample excluding participants with zero VILPA. Analyses adjusted for age, sex, duration of light intensity physical activity, duration of moderate intensity physical activity, smoking history, alcohol consumption, accelerometer estimated sleep duration, fruit and vegetable consumption, education, self-reported parental history of cardiovascular disease and cancer, and self-reported medication use (cholesterol, blood pressure, and diabetes), and prevalent cancer and CVD. All analyses were additionally adjusted for vigorous physical activity duration/frequency lasting more than one/two minute as appropriate.

B. Cardiovascular disease mortality

Frequency	Dose	HR	Lower 95 CI	Upper 95 CI
up to 1 min bout minimum dose	1.4	0.67	0.52	0.86
up to 1 min bout VILPA median	3.0	0.51	0.35	0.74
up to 1 min bout VILPA maximum	11.0	0.35	0.15	0.81
up to 2 min bout minimum dose	1.5	0.67	0.52	0.87
up to 2 min bout VILPA median	3.0	0.52	0.36	0.75
up to 2 min bout VILPA maximum	11.0	0.35	0.16	0.78
Duration	Dose	HR	Lower 95 CI	Upper 95 CI
up to 1 min bout minimum dose	3.4	0.73	0.58	0.91
up to 1 min bout VILPA median	4.4	0.66	0.50	0.88
up to 1 min bout VILPA maximum	16.0	0.45	0.29	0.72
up to 2 min bout minimum dose	4.1	0.72	0.57	0.91
up to 2 min bout VILPA median	4.4	0.68	0.52	0.89
up to 2 min bout VILPA maximum	16.0	0.45	0.30	0.69

Minimal dose (ED50 value): defined as the duration/frequency of VILPA associated with 50% of the optimal risk reduction. The VILPA duration and frequency median values were calculated in the sample excluding participants with zero VILPA. Analyses adjusted for age, sex, duration of light intensity physical activity, duration of moderate intensity physical activity, smoking history, alcohol consumption, accelerometer estimated sleep duration, fruit and vegetable consumption, education, self-reported parental history of cardiovascular disease and cancer, and self-reported medication use (cholesterol, blood pressure, and diabetes), and prevalent cancer. All analyses were additionally adjusted for vigorous physical activity duration/bouts lasting more than one/two minute as appropriate.

C. Cancer mortality

Frequency	Dose	HR	Lower 95 CI	Upper 95 CI
up to 1 min bout minimum dose	1.5	0.75	0.63	0.88
up to 1 min bout VILPA median	3.0	0.60	0.46	0.78
up to 1 min bout VILPA maximum	11.0	0.49	0.31	0.75
up to 2 min bout minimum dose	1.6	0.74	0.62	0.88
up to 2 min bout VILPA median	3.0	0.62	0.48	0.80
up to 2 min bout VILPA maximum	11.0	0.49	0.32	0.74
Duration	Dose	HR	Lower 95 CI	Upper 95 CI
up to 1 min bout minimum dose	3.4	0.76	0.66	0.87
up to 1 min bout VILPA median	4.4	0.70	0.59	0.84
up to 1 min bout VILPA maximum	16.0	0.51	0.38	0.69
up to 2 min bout minimum dose	4.1	0.74	0.63	0.85
up to 2 min bout VILPA median	4.4	0.70	0.60	0.83
up to 2 min bout VILPA maximum	16.0	0.50	0.38	0.66

Minimal dose (ED50 value): defined as the duration/frequency of VILPA associated with 50% of the optimal risk reduction. The VILPA duration and frequency median values were calculated in the sample excluding participants with zero VILPA. Analyses adjusted for age, sex, duration of light intensity physical activity, duration of moderate intensity physical activity, smoking history, alcohol consumption, accelerometer estimated sleep duration, fruit and vegetable consumption, education, self-reported parental history of cardiovascular disease and cancer, and self-reported medication use (cholesterol, blood pressure, and diabetes), and prevalent CVD. All analyses were additionally adjusted for vigorous physical activity duration/bouts lasting more than one/two minute as appropriate.

Supplementary Table 5: E-values for minimum dose, and median/maximum VILPA values for all-cause, cardiovascular disease mortality, and cancer mortality

A. All-cause mortality

Frequency	E-Value
up to 1 min bout minimum dose	2.01 (1.64)
up to 1 min bout VILPA median	2.66 (2.04)
up to 1 min bout VILPA maximum	3.26 (2.12)
up to 2 min bout minimum dose	1.95 (1.60)
up to 2 min bout VILPA median	2.61 (1.96)
up to 2 min bout VILPA maximum	3.26 (2.17)
Duration	
up to 1 min bout minimum dose	1.87 (1.54)
up to 1 min bout VILPA median	2.08 (1.63)
up to 1 min bout VILPA maximum	2.78 (2.04)
up to 2 min bout minimum dose	1.92 (1.56)
up to 2 min bout VILPA median	2.04 (1.63)
up to 2 min bout VILPA maximum	2.90 (2.17)

B. Cardiovascular disease mortality

Frequency	E-value
up to 1 min bout minimum dose	2.34 (1.60)
up to 1 min bout VILPA median	3.33 (2.04)
up to 1 min bout VILPA maximum	5.16 (1.77)
up to 2 min bout minimum dose	2.34 (1.58)
up to 2 min bout VILPA median	3.26 (2.00)
up to 2 min bout VILPA maximum	5.16 (1.88)
Duration	
up to 1 min bout minimum dose	2.10 (1.44)
up to 1 min bout VILPA median	2.40 (1.53)
up to 1 min bout VILPA maximum	3.87 (2.12)
up to 2 min bout minimum dose	2.14 (1.44)
up to 2 min bout VILPA median	2.30 (1.50)
up to 2 min bout VILPA maximum	3.87 (2.26)

C. Cancer mortality

Frequency	E-value
up to 1 min bout minimum dose	2.02 (1.53)
up to 1 min bout VILPA median	2.72 (1.88)
up to 1 min bout VILPA maximum	3.50 (2.00)
up to 2 min bout minimum dose	2.05 (1.53)
up to 2 min bout VILPA median	2.61 (1.81)
up to 2 min bout VILPA maximum	3.50 (2.04)
Duration	
up to 1 min bout minimum dose	1.97 (1.56)
up to 1 min bout VILPA median	2.21 (1.67)
up to 1 min bout VILPA maximum	3.33 (2.26)
up to 2 min bout minimum dose	2.04 (1.63)
up to 2 min bout VILPA median	2.21 (1.70)
up to 2 min bout VILPA maximum	3.41 (2.40)

Values represent: point estimate (and lower limit of the confidence interval in brackets) that an unmeasured confounder would need to have with both the exposure and outcome, conditional on the measured covariates to explain away the exposure-outcome association.

Supplementary Table 6: Hazard ratios associated with the minimum dose and median and maximum VPA values among exercisers for up to 2 minute bouts

All-cause mortality

Frequency	Dose	HR	Lower 95 CI	Upper 95 CI
up to 2 min bout minimum dose	1.7	0.76	0.70	0.82
up to 2 min bout VILPA median dose	4.0	0.59	0.51	0.68
up to 2 min bout VILPA maximum dose	14.0	0.53	0.43	0.65
Duration	Dose	HR	Lower 95 CI	Upper 95 CI
up to 2 min bout minimum dose	4.8	0.78	0.72	0.84
up to 2 min bout median VILPA dose	6.2	0.73	0.67	0.80
up to 2 min bout VILPA maximum dose	18.0	0.58	0.49	0.67

Cardiovascular disease mortality

Frequency	Dose	HR	Lower 95 CI	Upper 95 CI
up to 2 min bout minimum dose	1.6	0.63	0.54	0.73
up to 2 min bout VILPA median dose	4.0	0.39	0.29	0.52
up to 2 min bout VILPA maximum dose	14.0	0.27	0.16	0.46
Duration	Dose	HR	Lower 95 CI	Upper 95 CI
up to 2 min bout minimum dose	4.4	0.67	0.57	0.79
up to 2 min bout median VILPA dose	6.2	0.58	0.47	0.72
up to 2 min bout VILPA maximum dose	18.0	0.34	0.23	0.51

Cancer mortality

Frequency	Dose	HR	Lower 95 CI	Upper 95 CI
up to 2 min bout minimum dose	1.7	0.76	0.69	0.84
up to 2 min bout VILPA median dose	4.0	0.59	0.49	0.71
up to 2 min bout VILPA maximum dose	14.0	0.53	0.41	0.69
Duration	Dose	HR	Lower 95 CI	Upper 95 CI
up to 2 min bout minimum dose	5.0	0.77	0.70	0.85
up to 2 min bout median VILPA dose	6.2	0.73	0.65	0.82
up to 2 min bout VILPA maximum dose	18.0	0.55	0.44	0.67

Minimal dose (ED50 value): defined as the duration/frequency of VPA associated with 50% of the optimal risk reduction. The VPA duration and frequency median values were calculated in the sample excluding participants with zero VPA. Analyses adjusted for age, sex, duration of light intensity physical activity, duration of moderate intensity physical activity, smoking history, alcohol consumption, accelerometer estimated sleep duration, fruit and vegetable consumption, education, self-reported parental history of cardiovascular disease and cancer, and self-reported medication use (cholesterol, blood pressure, and diabetes), and prevalent cancer and CVD. All analyses were additionally adjusted for vigorous physical activity duration/frequency lasting more than two minutes.

Supplementary Table 7: Mean durations to reach vigorous intensity across five activities (unpublished data)

	%HR_{max}	%VO_{2max}	Rating of perceived exertion
Walking at a fast pace on a flat surface	76.2 (22.9) sec.	71.5 (18.9) sec	53.6 (19.7) sec
Walking at a fast pace carrying 5% of body weight	64.4 (32.4) sec	60.8 (25.2) sec	44.1 (23.7) sec
Walking at a fast pace carrying 10% of body weight	73.1 (34.6) sec	68.8 (21.3) sec	40.1 (20.3) sec
Walking at a fast pace on a 2.5% incline	85.4 (20.3) sec	83.0 (20.2) sec	66.8 (21.3) sec
Walking at a fast pace on a 7.0% incline	80.3 (28.0) sec	75.7 (15.8) sec	63.6 (21.9) sec

Supplementary Table 8: Intensity classification performance in 98 US and Australian adults

	Sensitivity	Specificity	Precision	F-score	Overall Accuracy	Weighted Kappa	Overall F-score
Sedentary	86.5	93.7	90.5	88.5			
Light	71.2	89.4	55.8	62.6			
Moderate	85.4	96.6	92.7	88.9			
Vigorous	95.4	99.4	94.6	95.0			
					84.6	0.78	83.8

Supplementary Table 9: Confusion matrix of activity classification in 98 US and Australian adults

	Sedentary	Light	Moderate	Vigorous
Sedentary	**36,904**	5,232	508	2
Light	3,120	**11,712**	1,612	17
Moderate	502	4,016	**29,528**	526
Vigorous	226	17	214	**9,470**
Rows= ground truth; columns=predictions; bold=correct labels; numbers represent each 10-second window; Derived from the US and Australian datasets				

Supplementary Table 10: STROBE Statement

	Item No	Recommendation	Page No
**Title and abstract**	1	(*a*) Indicate the study's design with a commonly used term in the title or the abstract	1, 2
		(*b*) Provide in the abstract an informative and balanced summary of what was done and what was found	2
**Introduction**			
Background/rationale	2	Explain the scientific background and rationale for the investigation being reported	3
Objectives	3	State specific objectives, including any prespecified hypotheses	3, 4
**Methods**			
Study design	4	Present key elements of study design early in the paper	17-19
Setting	5	Describe the setting, locations, and relevant dates, including periods of recruitment, exposure, follow-up, and data collection	17-19
Participants	6	(*a*) *Cohort study*—Give the eligibility criteria, and the sources and methods of selection of participants. Describe methods of follow-up<br>*Case-control study*—Give the eligibility criteria, and the sources and methods of case ascertainment and control selection. Give the rationale for the choice of cases and controls<br>*Cross-sectional study*—Give the eligibility criteria, and the sources and methods of selection of participants	17, Figure 1
		(*b*) *Cohort study*—For matched studies, give matching criteria and number of exposed and unexposed<br>*Case-control study*—For matched studies, give matching criteria and the number of controls per case	NA
Variables	7	Clearly define all outcomes, exposures, predictors, potential confounders, and effect modifiers. Give diagnostic criteria, if applicable	17-20
Data sources/ measurement	8*	For each variable of interest, give sources of data and details of methods of assessment (measurement). Describe comparability of assessment methods if there is more than one group	17-20

Bias	9	Describe any efforts to address potential sources of bias	19-20
Study size	10	Explain how the study size was arrived at	Figure 1
Quantitative variables	11	Explain how quantitative variables were handled in the analyses. If applicable, describe which groupings were chosen and why	19-20
Statistical methods	12	(*a*) Describe all statistical methods, including those used to control for confounding	19-20
		(*b*) Describe any methods used to examine subgroups and interactions	NA
		(*c*) Explain how missing data were addressed	17
		(*d*) *Cohort study*—If applicable, explain how loss to follow-up was addressed<br>*Case-control study*—If applicable, explain how matching of cases and controls was addressed<br>*Cross-sectional study*—If applicable, describe analytical methods taking account of sampling strategy	NA
		(*e*) Describe any sensitivity analyses	19-20
**Results**			
Participants	13*	(a) Report numbers of individuals at each stage of study—eg numbers potentially eligible, examined for eligibility, confirmed eligible, included in the study, completing follow-up, and analysed	Figure 1
		(b) Give reasons for non-participation at each stage	Figure 1
		(c) Consider use of a flow diagram	Figure 1
Descriptive data	14*	(a) Give characteristics of study participants (eg demographic, clinical, social) and information on exposures and potential confounders	Table 1
		(b) Indicate number of participants with missing data for each variable of interest	Figure 1
		(c) *Cohort study*—Summarise follow-up time (eg, average and total amount)	3
Outcome data	15*	*Cohort study*—Report numbers of outcome events or summary measures over time	3, Table 1, Figures 1-4
		Case-control study—Report numbers in each exposure category, or summary measures of exposure	*NA*

		Cross-sectional study—Report numbers of outcome events or summary measures	*NA*
Main results	16	(*a*) Give unadjusted estimates and, if applicable, confounder-adjusted estimates and their precision (eg, 95% confidence interval). Make clear which confounders were adjusted for and why they were included	3-6, Figures 2-4
		(*b*) Report category boundaries when continuous variables were categorized	NA
		(*c*) If relevant, consider translating estimates of relative risk into absolute risk for a meaningful time period	NA
Other analyses	17	Report other analyses done—eg analyses of subgroups and interactions, and sensitivity analyses	5-6
**Discussion**			
Key results	18	Summarise key results with reference to study objectives	6
Limitations	19	Discuss limitations of the study, taking into account sources of potential bias or imprecision. Discuss both direction and magnitude of any potential bias	7-8
Interpretation	20	Give a cautious overall interpretation of results considering objectives, limitations, multiplicity of analyses, results from similar studies, and other relevant evidence	6-8
Generalisability	21	Discuss the generalisability (external validity) of the study results	6, 8
**Other information**			
Funding	22	Give the source of funding and the role of the funders for the present study and, if applicable, for the original study on which the present article is based	8

Supplementary Figure 1: Flow diagram of exercisers

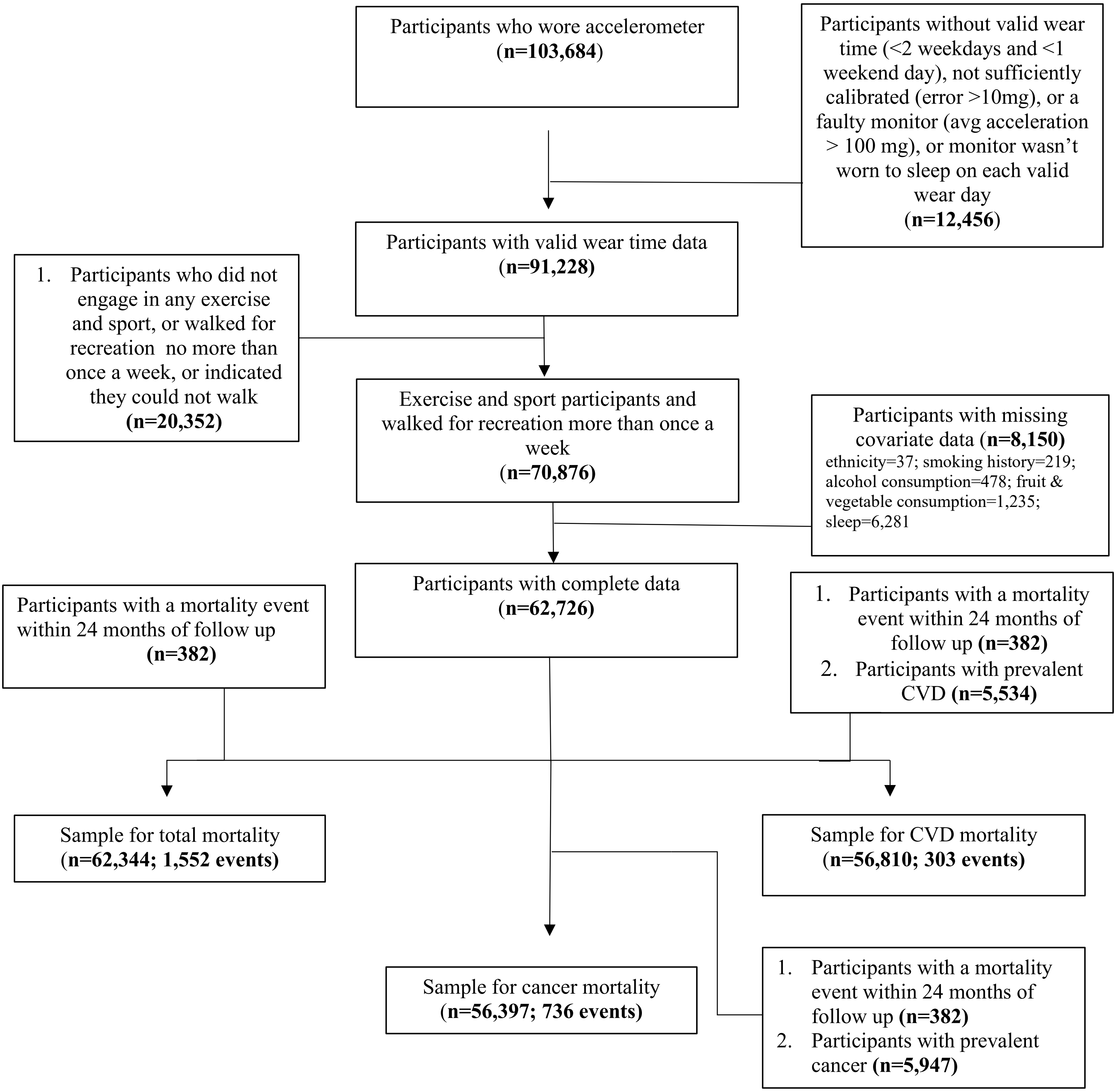

Supplementary Figure 2: Dose-response association of daily duration of vigorous intermittent lifestyle physical activity (VILPA) duration of bouts up to 2 minutes among those who reported no leisure time exercise and *no* recreational walking

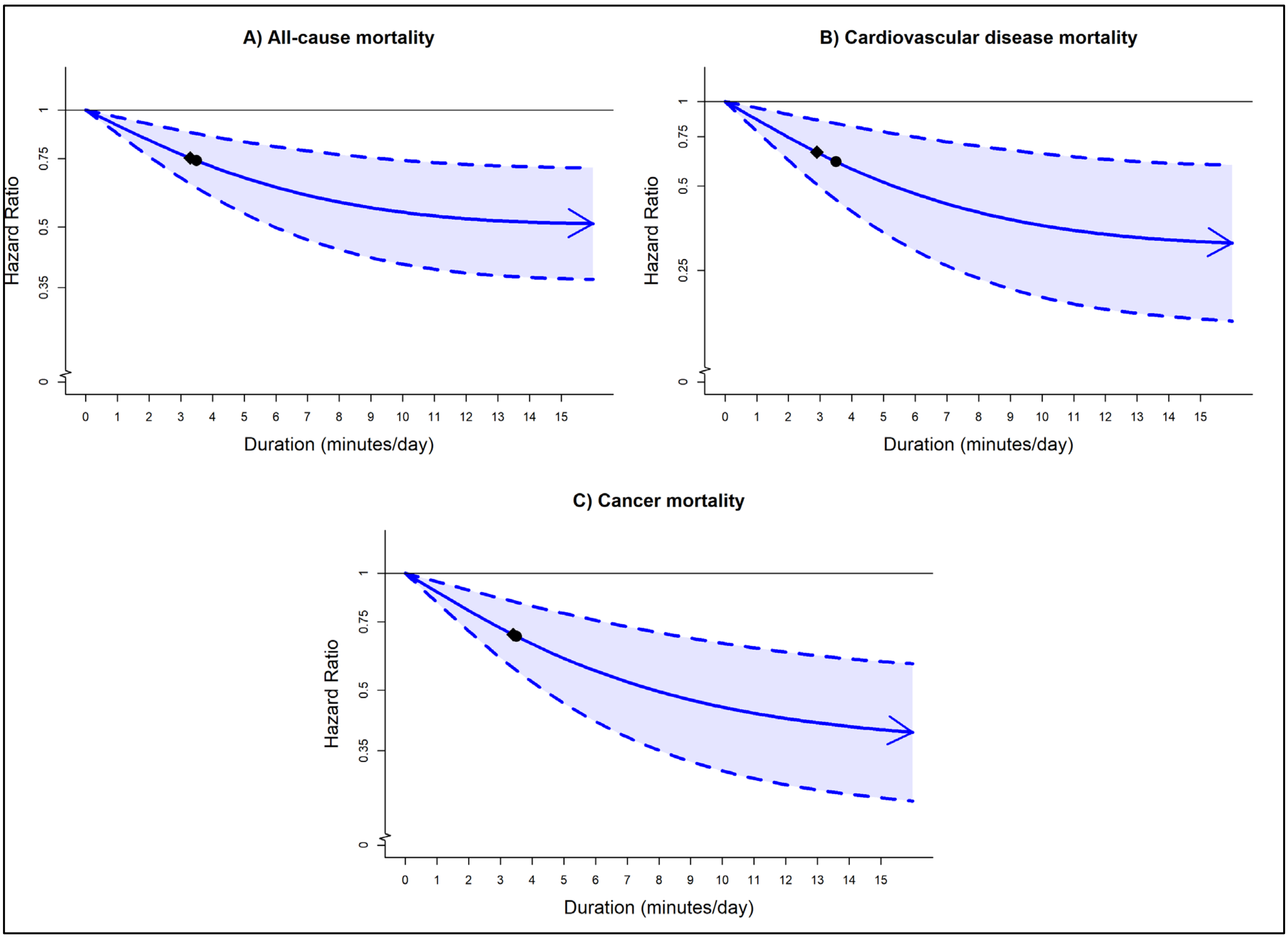

n= 10,230 were included in the all-cause mortality (411 events), n=10,161 in the CVD mortality (126 events) and n=10,092 in the cancer mortality analyses (226 events). Diamond: ED50 value, the minimal dose, defined as the duration of VILPA associated with 50% of the optimal risk reduction. Circle: effect associated with the median VILPA value. Analyses adjusted for age, sex, duration of light intensity physical activity, duration of moderate intensity physical activity, smoking history, alcohol consumption, accelerometer estimated sleep duration, fruit and vegetable consumption, education, self-reported parental history of cardiovascular disease and cancer, and self-reported medication use (cholesterol, blood pressure, and diabetes), and prevalent cancer and CVD (for all-cause mortality). All analyses were additionally adjusted for vigorous physical activity frequency of bouts lasting more than two minutes. See **Online Methods** for further details on the statistical methodology.

Supplementary Figure 3: Dose-response associations of energy expenditure-based volume of vigorous intermittent lifestyle physical activity (non-exercisers) and vigorous intensity physical activity (exercisers) with mortality (bouts lasting up to 2 minutes).

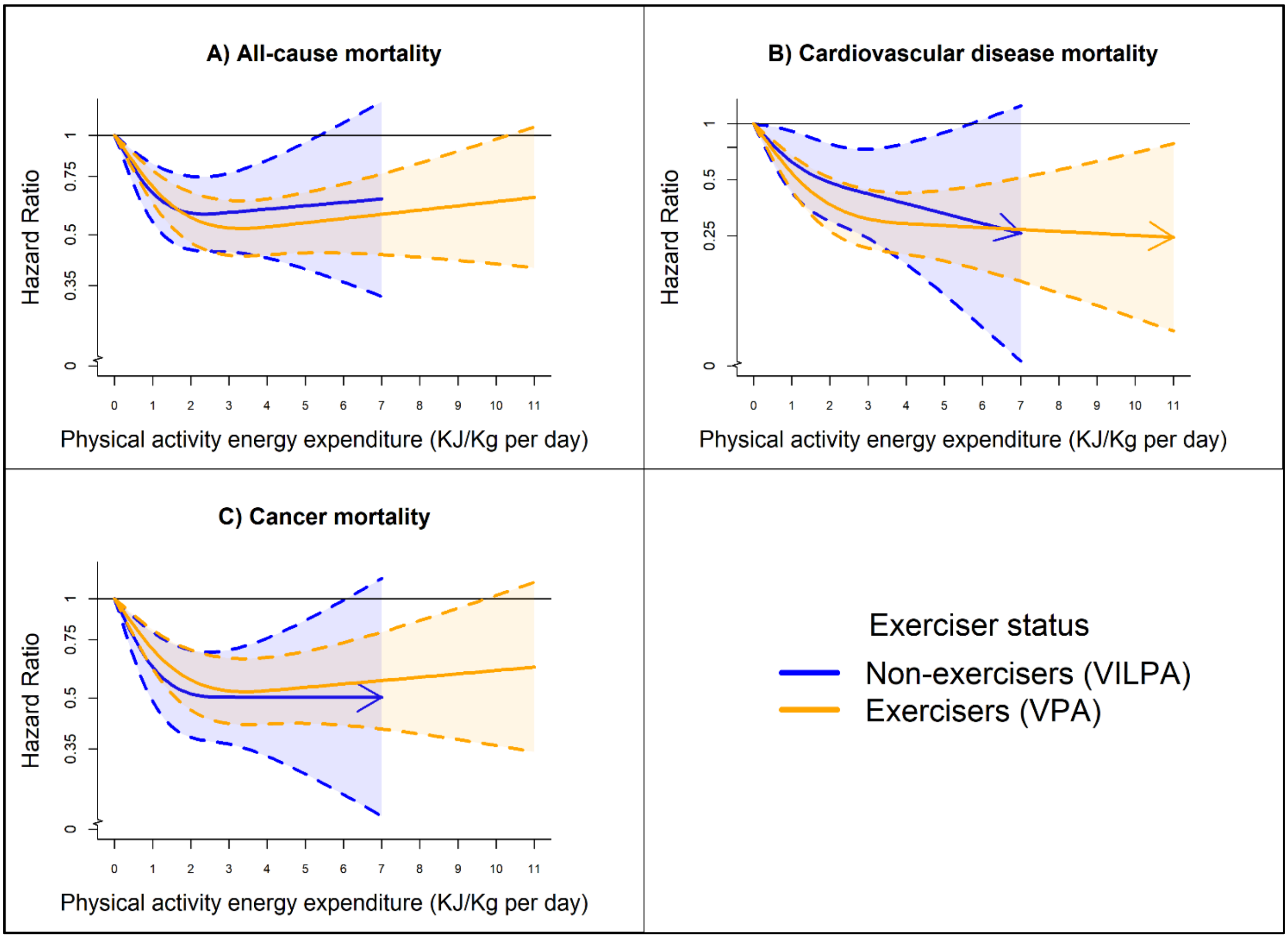

VILPA: vigorous intermittent lifestyle physical activity (non-exercisers); VPA: vigorous intensity physical activity (exercisers). Analyses adjusted for age, sex, duration of light intensity physical activity, duration of moderate intensity physical activity, smoking history, alcohol consumption, accelerometer estimated sleep duration, fruit and vegetable consumption, education, self-reported parental history of cardiovascular disease and cancer, and self-reported medication use (cholesterol, blood pressure, and diabetes), and prevalent cancer and CVD (for all-cause mortality). All analyses were additionally adjusted for vigorous physical activity frequency of bouts lasting more than two minutes. See Online Methods for further details on the statistical methodology.

Supplementary Figure 4: Physical activity type and intensity diagram

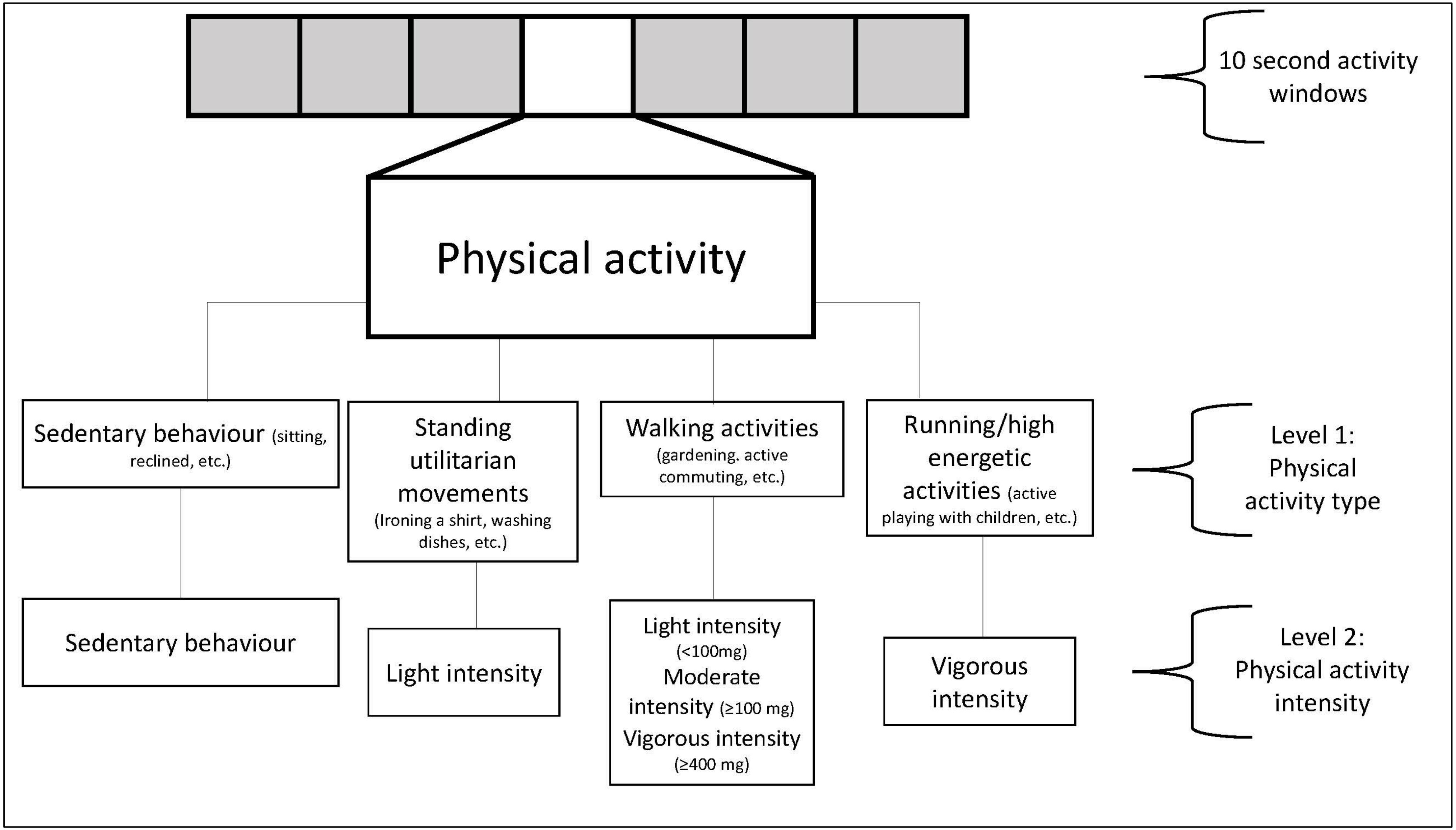

Supplementary Figure 5: Participant-level specific recall and precision of activity classification in 151 UK adults

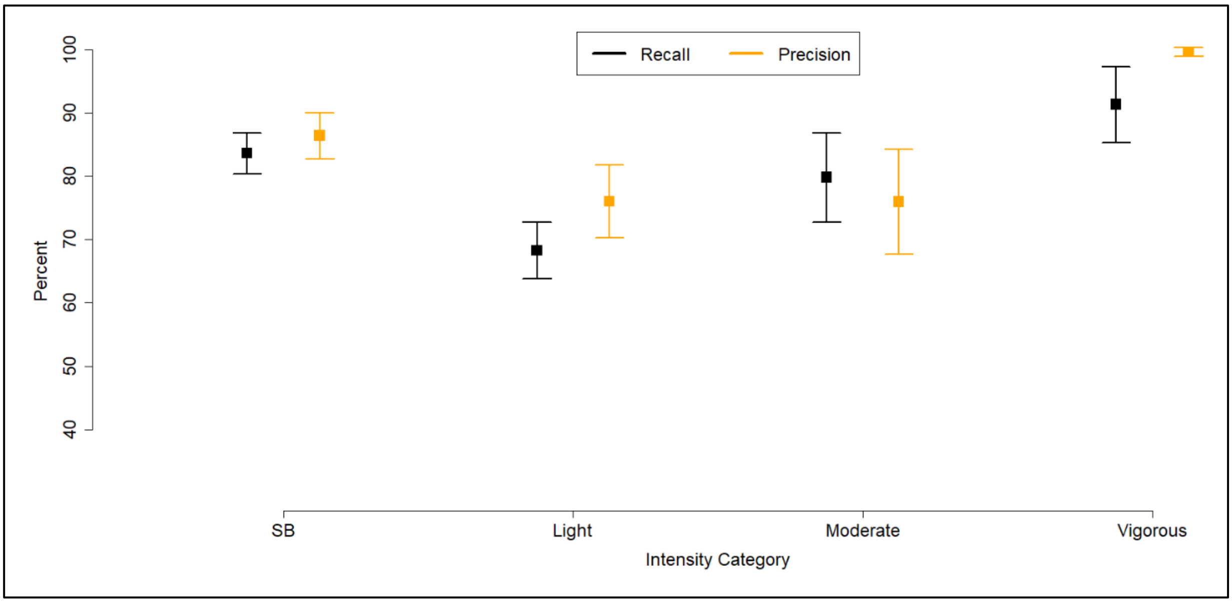

Supplement: Supplementary file 1 — Supplementary Tables 1–10 and Figs. 1–5. [file 41591_2022_2100_MOESM1_ESM.pdf]
