# Supplementary material for: Association of wearable device-measured vigorous intermittent lifestyle physical activity with mortality
Source: Nat Med. 2022 Dec 8;28(12):2521–9. doi: 10.1038/s41591-022-02100-x (PMC9800274; doi:10.1038/s41591-022-02100-x)
Supplement: Supplementary file 2 — Reporting Summary [file 41591_2022_2100_MOESM2_ESM.pdf]

nature portfolio

Corresponding author(s): Emmanuel Stamatakis

Last updated by author(s): Oct 19, 2022

Reporting Summary

Nature Portfolio wishes to improve the reproducibility of the work that we publish. This form provides structure for consistency and transparency in reporting. For further information on Nature Portfolio policies, see our Editorial Policies and the Editorial Policy Checklist.

Statistics

For all statistical analyses, confirm that the following items are present in the figure legend, table legend, main text, or Methods section.

n/a	Confirmed	
☐	☒	The exact sample size (n) for each experimental group/condition, given as a discrete number and unit of measurement
☐	☒	A statement on whether measurements were taken from distinct samples or whether the same sample was measured repeatedly
☒	☐	The statistical test(s) used AND whether they are one- or two-sided<br>*Only common tests should be described solely by name; describe more complex techniques in the Methods section.*
☐	☒	A description of all covariates tested
☐	☒	A description of any assumptions or corrections, such as tests of normality and adjustment for multiple comparisons
☐	☒	A full description of the statistical parameters including central tendency (e.g. means) or other basic estimates (e.g. regression coefficient) AND variation (e.g. standard deviation) or associated estimates of uncertainty (e.g. confidence intervals)
☒	☐	For null hypothesis testing, the test statistic (e.g. F, t, r) with confidence intervals, effect sizes, degrees of freedom and P value noted<br>*Give P values as exact values whenever suitable.*
☒	☐	For Bayesian analysis, information on the choice of priors and Markov chain Monte Carlo settings
☒	☐	For hierarchical and complex designs, identification of the appropriate level for tests and full reporting of outcomes
☐	☒	Estimates of effect sizes (e.g. Cohen's d, Pearson's r), indicating how they were calculated

Our web collection on statistics for biologists contains articles on many of the points above.

Software and code

Policy information about availability of computer code

Data collection: Data collection was undertaken elsewhere and this is clearly stated in the manuscript.

Data analysis: We performed all analysis using R statistical software with the RMS and survival packages, as stated in the manuscript: RMS package version 6.3.0; Survival package version 3.3.1; R version 4.2.1; Noldus Observer XT version 16.0

For manuscripts utilizing custom algorithms or software that are central to the research but not yet described in published literature, software must be made available to editors and reviewers. We strongly encourage code deposition in a community repository (e.g. GitHub). See the Nature Portfolio guidelines for submitting code & software for further information.

Data

Policy information about availability of data

All manuscripts must include a data availability statement. This statement should provide the following information, where applicable:

- Accession codes, unique identifiers, or web links for publicly available datasets
- A description of any restrictions on data availability
- For clinical datasets or third party data, please ensure that the statement adheres to our policy

Provide your data availability statement here.

Human research participants

Policy information about studies involving human research participants and Sex and Gender in Research.

Reporting on sex and gender	Sex was included as a covariate in the analyses. We were not able to provide sex-specific results due to low event numbers and dilution of statistical power. The UK Biobank collects information on the biological sex of participants and was self-reported.
Population characteristics	This study included a sample of 25,241 UK Biobank participants drawn from the general population with accelerometery data who reported no exercise in leisure time (mean age 62 years, 56% female, see Table 1). Over a mean follow-up of 6.9 (0.8) years 852 deaths deaths were recorded. Out of all VILPA bouts, 98% of them comprised bouts lasting up to two minutes. Sex was included as a covariate in the analyses.
Recruitment	A stratified approach was taken to the initial invites for the main UK Biobank Study with over-sampling of some age, gender and deprivation sub-groups. Participation was voluntary and the response rate was 5.5%. Invites to the accelerometry sub-study were sent randomly, although this was within the subset of participants that provided a valid email address. The response rate to the accelerometry sub-study was 44.8%. In the core analyses of the paper we used only non-exercisers: normally, wearable trackers cannot tell us whether an activity of a given intensity is done for exercise during leisure time or during daily living, making it practically impossible to examine domain-specific aspects of physical activity like VILPA. We used questionnaire-based leisure-time physical activity information in the UK Biobank to screen out exercisers, enabling us to determine VILPA participation. The resulting sample is not representative of the target population. The voluntary nature of participation, the low response rate, and the further selection pressure from the requirement to provide a valid email address means that this sample is not representative of the UK population. However, our group has produced empirical evidence showing that the low response rates and poor representativeness of the UK Biobank sample does not materially influence the associations between physical activity and mortality outcomes, see Stamatakis E et al. Epidemiology 2021 doi: 10.1097/EDE.0000000000001316
Ethics oversight	Ethical approval was covered under NHS National Research Ethics Service (Ref 11/NW/0382).

Note that full information on the approval of the study protocol must also be provided in the manuscript.

Field-specific reporting

Please select the one below that is the best fit for your research. If you are not sure, read the appropriate sections before making your selection.

☐ Life sciences ☒ Behavioural & social sciences ☐ Ecological, evolutionary & environmental sciences

For a reference copy of the document with all sections, see nature.com/documents/nr-reporting-summary-flat.pdf

Behavioural & social sciences study design

All studies must disclose on these points even when the disclosure is negative.

Study description	A prospective observational population-based study of quantitative UK Biobank data.
Research sample	UK Biobank Participants were drawn from the general population. This study included a sample of 25,423 UK Biobank participants with accelerometery data who reported no exercise in leisure time (mean age 62 years, 56% female, see Table 1). Over a mean follow-up of 6.9 (0.8) years 852 deaths deaths were recorded. Out of all VILPA bouts, 98% of them comprised bouts lasting up to two minutes.
Sampling strategy	A stratified approach was taken to the initial invites for the main UK Biobank Study with over-sampling of some age, gender and deprivation sub-groups. Participation was voluntary and the response rate was 5.5%. Invites to the accelerometry sub-study were sent randomly, although this was within the subset of participants that provided a valid email address. The response rate to the accelerometry sub-study was 44.8%. In the core analyses of the paper we used only non-exercisers: normally, wearable trackers cannot tell us whether an activity of a given intensity is done for exercise during leisure time or during daily living, making it practically impossible to examine domain-specific aspects of physical activity like VILPA. We used questionnaire-based leisure-time physical activity information in the UK Biobank to screen out exercisers, enabling us to determine VILPA participation. The resulting sample is not representative of the target population.
Data collection	The main exposure measure of wearable device measured physical activity was collected using a wrist-worn triaxial accelerometer. This was sent to the participant with instructions about how to wear it for seven days, before returning the monitor. The majority of the covariate data including demographic, other lifestyle, and health status were collected via a touchscreen self-administered questionnaire completed at assessment centres. Anthropometric measures were also obtained at these visits. Health status data were supplemented using linked hospital episode statistics. Sample size calculations are not appropriate for this kind of research and were not performed, we used maximum sample available meeting the key eligibility criteria of being a non-exerciser and having valid data across all variables used in these analyses. The resultant sample size of n=25,241 is sufficient for this kind of analyses, as indicated by analogous recent manuscripts in Nature Medicine, eg. n=6,042 in Master, H., Annis, J., Huang, S. et al. Association of

	step counts over time with the risk of chronic disease in the All of Us Research Program. Nat Med (2022). https://doi.org/10.1038/s41591-022-02012-w
Timing	The physical activity accelerometry measurement occurred between 2013 and 2016. This was a median of 6.9 years after the baseline recruitment assessment centre visits that occurred between 2006 and 2010. A minority of participants undertook follow-up assessment centre visits in the period 2008 to 2018. Covariate data from the visit closest to the physical activity measurement were used.
Data exclusions	Out of the 103,695 with accelerometry data, 7000 were excluded as they did not pass the quality control procedures. We also excluded from the core analyses those who died within the first 2 years of follow up, and those who reported participation in any leisure time exercise or recreational walking more than once a week. In a additional analysis we analysed the 62,344 UK Biobank exercisers (1,552 deaths).
Non-participation	The response rates for the main UK Biobank Study and accelerometer sub-study were 5.5% and 44.8% respectively, as described in the sampling strategy. Follow-up was based on mortality records and hospital admissions so there was no loss to follow up that we were aware of.
Randomization	This was an observational study and so there was no randomisation. Covariate selection was made a priori, based on previous literature with the aim of causally inferring the relationship between physical activity and mortality. Based on our assumptions of how the covariates influence the relationship under study, we grouped them into those potentially on the causal pathway, and those not on the causal pathway. We performed statistical models that progressively adjusted for these two groups of covariates.

Reporting for specific materials, systems and methods

We require information from authors about some types of materials, experimental systems and methods used in many studies. Here, indicate whether each material, system or method listed is relevant to your study. If you are not sure if a list item applies to your research, read the appropriate section before selecting a response.

Materials & experimental systems

n/a	Involved in the study
☒	☐ Antibodies
☒	☐ Eukaryotic cell lines
☒	☐ Palaeontology and archaeology
☐	☒ Animals and other organisms
☒	☐ Clinical data
☒	☐ Dual use research of concern

Methods

n/a	Involved in the study
☒	☐ ChIP-seq
☒	☐ Flow cytometry
☒	☐ MRI-based neuroimaging

Animals and other research organisms

Policy information about studies involving animals; ARRIVE guidelines recommended for reporting animal research, and Sex and Gender in Research

Laboratory animals	N/A
Wild animals	N/A
Reporting on sex	N/A
Field-collected samples	N/A
Ethics oversight	N/A

Note that full information on the approval of the study protocol must also be provided in the manuscript.
